# Chitosan-quinoxaline Schiff base hydroxyapatite composite with antimicrobial properties for bone regeneration

**DOI:** 10.1038/s41598-026-61289-w

**Published:** 2026-07-15

**Authors:** Abdelrahman Barakat, Gameel A. M. Ehagali, Shahira H. EL-Moslamy, Moustafa S. Abusaif, Medhat E. Owda, Yousry A. Ammar, Mohamed B. Ghazy

**Affiliations:** 1 Department of Chemistry, Faculty of Science (boys), Al-Azhar University, Nasr City, Cairo, 11884 Egypt; 2 Bioprocess Development Department (BID), Genetic Engineering and Biotechnology Research Institute (GEBRI), City of Scientific Research and Technological Applications (SRTA-City), New Borg El-Arab City, 21934 Alexandria Egypt; 3 Department for Synthesis and Characterization of Polymers, Polymer Institute of the Slovak Academy of Sciences, Dúbravská cesta 9, Bratislava, 845 41 Slovak Republic

**Keywords:** Chitosan Schiff base, Hydroxyapatite, Bone regeneration, Biotechnology, Chemistry, Materials science, Microbiology

## Abstract

Implant-associated infections remain a major challenge in bone regeneration, often compromising the long-term success of hydroxyapatite-based biomaterials. A new sulfonated quinoxaline aldehyde, 2-ethoxy-4-formylphenyl 2,3-dioxo-1,2,3,4-tetrahydroquinoxaline-6-sulfonate (B6), was synthesized and covalently grafted onto chitosan via Schiff base condensation to yield derivatives with controlled degrees of substitution (d1–d4). Which were subsequently incorporated into hydroxyapatite to form composite systems (dH0–dH4). Structural characterization using Fourier-transform infrared spectroscopy (FTIR), nuclear magnetic resonance (NMR), X-ray diffraction (XRD), and X-ray photoelectron spectroscopy (XPS) confirmed successful Schiff base formation, while thermogravimetric analysis (TGA) indicated enhanced thermal stability. Scanning electron microscopy with energy-dispersive X-ray analysis (SEM–EDX) and Brunauer–Emmett–Teller (BET) analyses revealed homogeneous hydroxyapatite dispersion and a mesoporous architecture (44.8 m^2^ g^− 1^, ~ 20 nm pores) for the optimized composite. Among the investigated formulations, dH2 exhibited the most balanced physicochemical and biological performance. The dH2 composite exhibited pronounced antimicrobial activity, achieving biofilm inhibition of 89.65% against *Bacillus cereus* and 86.65% against *Staphylococcus aureus*, with MIC values lower than those of reference antibiotics under the tested conditions. Cytotoxicity assessment using Vero and MG-63 cells demonstrated excellent biocompatibility, with > 99% cell viability at ≤ 125 µg/mL and IC_50_ values of 344.99 and 156.62 µg/mL, respectively. These findings indicate that the developed chitosan–quinoxaline Schiff base hydroxyapatite composite represents a multifunctional, infection-resistant platform suitable for bone regeneration applications.

**Supplementary Information:**

The online version contains supplementary material available at 10.1038/s41598-026-61289-w.

## Introduction

Bone defects caused by trauma or disease pose major clinical challenges, especially in large or irregular defects. Although autologous grafts remain the gold standard, limitations such as donor-site morbidity and limited availability drive the demand for effective synthetic bone substitutes^1,2^.

Among calcium phosphate-based materials, hydroxyapatite (HAp, Ca_10_(PO_4_)_6_(OH)_2_) is widely used due to its chemical similarity to bone and excellent biocompatibility. However, its brittleness, low mechanical strength, and lack of antimicrobial properties limit its standalone use in bone repair^3^. To overcome these limitations, composite systems combining HAp with organic polymers have been extensively explored to improve overall structural and functional performance^4,5^. In the context of bone tissue engineering, the design of biomimetic scaffolds requires careful consideration of key parameters, including interconnected porosity, mechanical integrity, osteoconductivity, and biological functionality^6^. Polymer–ceramic composites have gained increasing attention due to their ability to mimic the hierarchical structure of natural bone while providing enhanced mechanical properties and bioactivity. Recent advances have focused on the development of multifunctional scaffolds capable of simultaneously supporting bone regeneration and preventing microbial colonization. Such hybrid systems can be further enhanced through surface modification and incorporation of bioactive moieties to improve cell adhesion, proliferation, and antibacterial performance^7,8^.

Natural polymers such as collagen, alginate, cellulose, and chitosan offer superior biocompatibility compared to synthetic counterparts. Among them, chitosan stands out due to its biodegradability, amino-rich structure, and inherent antimicrobial activity^9–11^.

Chemical modification of chitosan through Schiff base formation introduces imine (C = N) linkages that can effectively alter its physicochemical behavior. Such modifications often improve solubility, thermal stability, and the ability of chitosan to interact with biological systems. Importantly, Schiff base derivatives commonly display enhanced antimicrobial activity due to the incorporation of electron-rich aldehyde functionalities, making them attractive candidates for use in polymer–ceramic composite biomaterials^12^. Such chemical modifications are particularly important in biomedical applications, where tuning the surface chemistry of chitosan can directly influence cell adhesion, proliferation, and antimicrobial performance^13^.

Quinoxalines represent a structurally versatile class of heterocyclic compounds with well-documented antimicrobial and therapeutic properties^14^. Their framework can be readily functionalized to introduce bioactive groups with targeted activity. When used as aldehyde precursors, quinoxalines readily form stable Schiff bases with chitosan, producing polymeric derivatives that combine the biological activity of the quinoxaline core with the biocompatibility and film-forming capability of chitosan. These hybrid systems have demonstrated favorable interactions with inorganic phases such as hydroxyapatite, supporting their use in advanced biomedical composites^15^. Beyond their antimicrobial activity, quinoxaline derivatives can play a key role in bone-related applications by introducing electron-rich and heteroatom-containing functional groups that enhance intermolecular interactions with calcium phosphate phases. Such interactions can enhance interfacial compatibility, stability, and biological performance of hydroxyapatite-based composites. Therefore, the incorporation of quinoxaline moieties into polymeric scaffolds represents a promising strategy for designing multifunctional biomaterials for bone tissue regeneration. In addition, the incorporation of quinoxaline derivatives into scaffold systems may contribute to improved cell–material interactions and enhanced biological performance relevant to bone tissue engineering applications, making them particularly attractive for bone tissue engineering applications^16^.

However, to date, no study has reported the integration of sulfonated quinoxaline-based chitosan Schiff bases into hydroxyapatite matrices with simultaneous evaluation of structural, antimicrobial, and cytocompatibility performance. In the present work, a new sulfonated quinoxaline aldehyde (B6) was synthesized and used to prepare a series of chitosan Schiff base derivatives with controlled substitution levels. Incorporating this engineered heterocycle into the chitosan backbone was intended to introduce additional bioactive and electronically functional groups that could improve the overall biological performance of the resulting materials. These derivatives were subsequently integrated with hydroxyapatite to generate hybrid composites with enhanced antimicrobial function and biological compatibility.

To address the intrinsic limitations of hydroxyapatite (HAp) in bone regeneration—particularly its weak antimicrobial activity and modest biological response—this study proposes the development of a new HAp-based composite incorporating a quinoxaline-functionalized chitosan Schiff base. The central rationale is that introducing a sulfonated quinoxaline moiety into the chitosan backbone will provide additional bioactivity, improve compatibility with HAp, and reinforce the overall structural performance of the composite. A combination of FTIR, NMR, MS, XRD, SEM, BET surface analysis, and TGA was employed to elucidate the molecular structure of the synthesized Schiff base and confirm its incorporation into the HAp framework. These techniques were also used to evaluate crystallinity, surface morphology, and the thermal and textural properties of the resulting composites. In addition, compressive mechanical testing was performed to assess the structural reliability of the prepared scaffolds, ensuring their suitability for non-load-bearing bone regeneration applications. In parallel, biological assessments were performed to evaluate the functional performance of the composites. Antimicrobial activity was examined against clinically relevant pathogens, while cytotoxicity and biocompatibility were evaluated using both Vero cells and MG-63 osteoblast-like cells to capture early cellular response.

Overall, this work introduces a practical strategy for engineering multifunctional HAp composites by incorporating chitosan Schiff bases derived from a newly synthesized quinoxaline aldehyde. The resulting materials combine antibacterial activity with favorable cytocompatibility, highlighting their potential for advanced biomedical applications in bone tissue engineering.

## Materials and methods

### Materials

Chitosan (85% deacetylated, ~ 4 × 10^5^ g/mol) was obtained from Alfa Aesar (USA). *o*-Phenylenediamine, chlorosulfonic acid (ClSO_3_H), and ethyl vanillin were purchased from Sigma–Aldrich (Germany). Hydrochloric acid, sulfuric acid, oxalic acid, K_2_HPO_4_, CaCl_2_, KOH, and NaOH were supplied by El Nasr Company (Cairo, Egypt). Analytical-grade solvents—acetone, DMSO, DMF, ethanol, toluene, THF, and acetic acid—were purchased from Fluka (Germany).

The antimicrobial assay was conducted using clinically relevant pathogens: two Gram-positive strains (*Staphylococcus epidermidis* ATCC 14990 and *Staphylococcus aureus* ATCC 25923), two Gram-negative strains (*Salmonella paratyphi* ATCC 9150 and *Escherichia coli* ATCC 10536), and two yeast strains (*Candida glabrata* ATCC 66032 and *Candida albicans* ATCC 10231). All microbial strains were supplied by the Bioprocess Development Department, Genetic Engineering and Biotechnology Research Institute, SRTA-City (Alexandria, Egypt).

Vero cells (normal kidney epithelial cells derived from African green monkey, *Cercopithecus aethiops*) and MG-63 cells (human osteosarcoma-derived osteoblast-like cells) were used for cytotoxicity assessment. Vero cells were cultured in Roswell Park Memorial Institute medium (RPMI-1640) medium, whereas MG-63 osteoblast-like cells were grown in Dulbecco’s Modified Eagle Medium (DMEM); both media were supplemented with 10% fetal bovine serum and 1% penicillin–streptomycin. All cell culture reagents, including PBS, 96-well plates, and MTT solution (5 mg/mL in PBS), were obtained from BIO BASIC CANADA INC.

### Methods

#### Preparation of 2-ethoxy-4-formylphenyl 2,3-dioxo-1,2,3,4-tetrahydroquinoxaline-6-sulfonate (B6)

The target molecule B6 was synthesized through a three-step sequence involving intermediates B0 and B1.

Step 1: Synthesis of 2,3-(1 H,4 H)-quinoxalinedione (B0)

*o*-Phenylenediamine and oxalic acid were refluxed in 4 N HCl for 4 h following the procedure reported in literature^17^. The precipitated solid was filtered, washed, and dried to obtain pure B0.

Step 2: Preparation of 2,3-dioxo-1,2,3,4-tetrahydroquinoxaline-6-sulfonyl chloride (B1)

B0 (5.0 g, 3.09 mmol) was gradually added to chlorosulfonic acid at room temperature to control the exothermic reaction. The mixture was refluxed at 110 °C for 8 h, cooled, and poured onto crushed ice. The resulting solid was filtered, washed with cold water, dried, and recrystallized from dry toluene–acetone to obtain B1 (M.p. 330 °C, 85% yield)^15^.

Step 3: Synthesis of the quinoxaline aldehyde derivative B6

B1 (1 mmol, 2.6 g) was dissolved in 20 mL DMF and refluxed with ethyl vanillin (1 mmol, 1.67 g) for 3 h, then stirred at room temperature for 2 additional hours. The product was filtered and recrystallized from ethanol/DMF to yield off-white crystals of B6 (M.p. 290–292 °C, yield 90%).

#### Synthesis of chitosan Schiff bases

A solution of chitosan (1.0 g, 0.55 mmol) was prepared by dissolving it in 100 mL of 1% (v/v) acetic acid at ambient temperature. Separately, B6 was dissolved in 25 mL ethanol with traces of acetic acid and added dropwise to the chitosan solution under continuous stirring. The mixture was heated at 70 °C for 24 h to promote Schiff base formation. The reaction product was precipitated using excess ethanol, neutralized with 0.1 N NaOH, filtered, washed with ethanol to remove unreacted aldehyde, and finally rinsed with acetone. Different B6-to-chitosan molar ratios (1:1, 0.75:1, 0.5:1, and 0.25:1 mmol/g) were employed to prepare a series of Schiff base derivatives and coded as shown in Table 1.

**Table 1:** Sample codes of chitosan-B6 Schiff bases according to B6: chitosan molar ratios.

Chitosan-B6 Schiff base code	Quinoxaline aldehyde derivative (B6) (mol)	Chitosan (mol)
d1	1 × 10^− 2^	0.55 × 10^− 2^
d2	0.75 × 10^− 2^	0.55 × 10^− 2^
d3	0.5 × 10^− 2^	0.55 × 10^− 2^
d4	0.25 × 10^− 2^	0.55 × 10^− 2^

#### Preparation of hydroxyapatite composites with chitosan and chitosan-B6 Schiff bases

Composites were prepared using unmodified chitosan and the synthesized Schiff bases (d1–d4). A composite with unmodified chitosan served as a control to assess the impact of Schiff base functionalization on composite integration and performance. The mineralization process was carried out at a polymer concentration of 2 mg/mL using chitosan and the synthesized chitosan-Schiff bases (d1, d2, d3, and d4). A 50 mL polymer solution containing CaCl_2_ (0.48 M) was added dropwise to 50 mL of polymer solution containing K_2_HPO_4_ (0.24 M) under stirring. The pH was adjusted to 8 using 0.1 M KOH, and the mixture was stirred overnight at room temperature. The resulting precipitate was collected by centrifugation (1500 rpm, 20 min), washed with deionized water, and dried under vacuum for 48 h^18^. The composites were coded as dH0–dH4 (Table 2), where dH0 contains unmodified chitosan, and dH1–dH4 correspond to Schiff base derivatives d1–d4.

**Table 2:** Codes and descriptions of hydroxyapatite composites prepared with chitosan and chitosan-Schiff bases.

Sample code	Polymer	Composite description
dH0	Chitosan	Chitosan-HAp composite
dH1	d1	d1- HAp composite
dH2	d2	d2- HAp composite
dH3	d3	d3- HAp composite
dH4	d4	d4- HAp composite

### Measurements

#### Assessment of degree of substitution

The nitrogen content (N%) of native chitosan and its Schiff base derivatives was quantified using the Kjeldahl method, and the degree of substitution (DS) was calculated using Eq. 1^19^.

1 $$\:\:\:\:\:\:\:\:\:\:\:\:\:\:\:\:\:\:\:\:DS=1-\left(\frac{M2.N2}{M1.N1}\right)$$

Here, *M₁* and *M₂* refer to the molar amounts of substituted chitosan and the glucosamine monomer, respectively, while *N₁* and *N₂* correspond to the nitrogen contents of the Schiff base and the unmodified chitosan.

#### Ion exchange capacity (ICE)

IEC was measured to assess changes in proton-binding sites introduced by Schiff base modification. Samples (d1–d4) were equilibrated in 0.1 M H₂SO₄ for 12 h, filtered, and the remaining acid was titrated with 0.1 N NaOH. A blank titration (without sample) was also performed as a reference. IEC was determined using Eq. 2.

2 $$\:\:\:\:\:\:\:\:\:\:\:\:\:\:\:\:\:\:\:\:\:\:\:\:\:\mathrm{I}\mathrm{E}\mathrm{C}(\:\mathrm{m}\mathrm{e}\mathrm{q}/\mathrm{g})=\frac{\left({\mathrm{V}}_{\mathrm{b}}-{\mathrm{V}}_{\mathrm{a}}\right)\:\mathbf{N}}{\mathbf{W}}$$

Here, *V*_*a*_ and *V*_*b*_ represent the volumes of NaOH used for the blank and the sample, respectively; *N* is the normality of the NaOH solution, and *W* denotes the weight of the sample^20^.

#### Solubility test

Chitosan and its derivatives were evaluated for solubility at a concentration of 10 mg/mL in different solvents (distilled water, 1% acetic acid, acetone, DMSO, methanol, DMF, and THF) at 25 °C. Each sample was placed in a sealed test tube containing the solvent and left undisturbed for 24 h before assessing solubility.

### Instrumental characterization

*FTIR*: Fourier-transform infrared spectra of B6, chitosan Schiff bases, and HAp composites (dH0–dH4) were obtained using a Shimadzu FTIR-8400 S (4000–400 cm^− 1^, 16 scans). FTIR was used to confirm imine formation and to verify HAp incorporation by identifying characteristic phosphate vibrations.

^1^*H and* ^13^*C NMR*: NMR spectra for B6 and its chitosan Schiff base were recorded using a JEOL JNM-ECA 500 II spectrometer, operating at 500 MHz for ^1^H and 126 MHz for ^13^C. DMSO-*d*_*6*_ was used as the primary NMR solvent. For partially soluble derivatives, a small amount of TFA-*d* was added to facilitate dissolution and obtain spectra of sufficient quality for structural characterization.

*Mass spectra (MS)*: The B6 compound was analyzed via electron impact mass spectrometry using a Shimadzu GC–2010 system. Ionization was performed at 70 eV, with the ion source and interface temperatures set at 230 °C and 250 °C, respectively. Helium served as the carrier gas at a flow rate of 1.0 mL/min.

*X-ray diffraction analysis (XRD)*: XRD measurements were carried out on a PW 1830 diffractometer (Japan) using Cu-Kα radiation (λ = 1.5406 Å), over a 2θ range of 5°–60°. The crystallinity index (CI) was calculated using Eq. 3^21^.

3 $$C_{I} = \frac{{I110 - Iam}}{{I110}} \times 100\user2{\% }$$

Where *I₁₁₀* is the peak intensity at 2θ (crystalline region), and *I*_*am*_ is the intensity of the amorphous region at 15.7°. Full width at half maximum (FWHM) of the XRD peaks was determined by peak fitting using Gaussian functions in OriginPro software, and these values were subsequently used to calculate crystallite sizes of HAp composites *via* Scherrer’s equation: $\:D=\frac{K\lambda\:}{\beta\:{cos}\theta\:}$

Where *D* is crystallite size (nm), *K* = 0.89, *λ* = 1.5406 Å, *β* is the full width at half maximum (FWHM, in radians), and *θ* is the Bragg angle^22^.

The unit cell parameters were refined from the diffraction data using the cell refinement module of HighScore Plus software. lattice parameters (a, b, c, α, β, γ), unit cell volume, and space group were automatically calculated from the refined diffraction peaks.

The reliability of the refinement was verified by matching the calculated and experimental peak positions, confirming the hexagonal P6_3_/mmc crystal symmetry.

The Reference Intensity Ratio (RIR) values were obtained from the internal software database for phase quantification.

*X-ray photoelectron spectroscopy (XPS)*: The surface elemental composition and chemical states were examined by X-ray photoelectron spectroscopy (XPS) using an ESCALAB 250Xi system (Thermo Scientific, USA).

*Thermogravimetric Analysis (TGA)*: Thermal stability was evaluated using a TGA instrument (SDT Q600 V20.9 Build 20), heating samples at 10 °C min^− 1^ up to 600 °C under nitrogen flow (5 mL min^− 1^).

*Scanning electron microscope (SEM)*: Surface morphologies of chitosan and its Schiff base derivatives (d1–d4) were imaged using a JEOL JSM-6360LA SEM at 2000×, 4000×, and 16,000× magnifications. For composites (dH0–dH4), higher magnifications (4000×, 13000×, 25000×, and 100000×) were used to assess surface features. Accelerating voltages of 10 and 30 kV were applied depending on sample and magnification.

*BET Surface Area and Porosity Analysis*: Textural properties of the prepared composite (dH2) were determined using N_2_ adsorption–desorption isotherms recorded at 77 K on a BELSORP-miniX surface area and porosimeter analyzer (BEL Japan Inc.). Prior to analysis, samples were degassed under vacuum at 110 °C for 12 h. The specific surface area was calculated using the Brunauer–Emmett–Teller (BET) equation within the linear region of the isotherm (P/P₀ = 0.05–0.22). Pore size distribution was obtained from the adsorption and desorption branches using the Barrett–Joyner–Halenda (BJH) method based on the Harkins–Jura standard thickness curve. Total pore volume was determined at P/P₀ = 0.99, while average pore diameter was calculated from the desorption branch.

*Energy Dispersive X-ray (EDX)*: EDX analysis, performed alongside SEM at an accelerating voltage of 30 kV, was used to assess the elemental composition and determine the Ca/P ratios of the prepared composites (dH0–dH4).

*Mechanical properties*: Cylindrical specimens (10 mm diameter × 20 mm height) of the chitosan/HAp composite (dH0) and the Schiff base–modified composite (dH2) were prepared by casting the hydrated mixtures into Teflon molds, followed by drying at 40 °C for 48 h until complete solidification. Mechanical compression tests were performed using a Zwick/Roell universal testing machine (Zwick/Roell GmbH, Germany) equipped with a 10 kN load cell. A preload of 0.5 N was applied to ensure full contact between the specimen and compression plates, after which samples were compressed at a constant crosshead speed of 5 mm min^− 1^ in accordance with ASTM D2659. Force–displacement data were recorded using the TestXpert II software, which was used to calculate the compressive strength (Fmax). Stress–strain curves were generated automatically, and Young’s modulus (E) was obtained from the linear elastic region.

### Antimicrobial bioassay

The antimicrobial properties of the synthesized hydroxyapatite–chitosan composites (dH0–dH4) were systematically evaluated to investigate the effect of Schiff base functionalization on antibacterial and antifungal performance. To obtain a comprehensive assessment, three complementary assays were conducted: a biofilm inhibition assay to assess the ability of the materials to prevent microbial adhesion and growth, a time-kill kinetics study to quantify their bactericidal activity over time, and MIC/MBC/MFC determinations to establish the minimal effective concentrations of the most active formulation.

#### In vitro biofilm inhibition assay

Biofilm inhibition was quantified according to the National Committee for Clinical Laboratory Standards (NCCLS) broth microdilution guidelines^23^. All tested microbial strains are recognized biofilm-forming pathogens capable of adhering to abiotic surfaces and producing structured biofilms under in vitro conditions^24^. Biofilms were formed in sterile 96-well polystyrene microplates. Microbial suspensions (2 × 10^5^ CFU/mL) were added to well containing 100 µL of composite under aseptic conditions and incubated at 37 °C with shaking (200 rpm) for 24–48 h. Untreated cultures served as negative controls. After incubation, wells were gently washed three times with sterile phosphate-buffered saline (PBS) to remove non-adherent cells. Optical density (OD) at 600 nm was recorded, and the percentage inhibition was calculated using Eq. (4). This assay provided a direct measure of each composite’s ability to prevent biofilm formation^25^.

4 $$\:\mathbf{P}\mathbf{e}\mathbf{r}\mathbf{c}\mathbf{e}\mathbf{n}\mathbf{t}\mathbf{a}\mathbf{g}\mathbf{e}\:\mathbf{o}\mathbf{f}\:\mathbf{b}\mathbf{i}\mathbf{o}\mathbf{f}\mathbf{i}\mathbf{l}\mathbf{m}\:\mathbf{i}\mathbf{n}\mathbf{h}\mathbf{i}\mathbf{b}\mathbf{i}\mathbf{t}\mathbf{i}\mathbf{o}\mathbf{n}=\left[\frac{\left({\mathrm{O}\mathrm{D}}_{\mathrm{u}\mathrm{n}\mathrm{t}\mathrm{r}\mathrm{e}\mathrm{a}\mathrm{t}\mathrm{e}\mathrm{d}\:}-{\mathrm{O}\mathrm{D}}_{\mathrm{t}\mathrm{r}\mathrm{e}\mathrm{a}\mathrm{t}\mathrm{e}\mathrm{d}}\right)}{{\mathrm{O}\mathrm{D}}_{\mathrm{u}\mathrm{n}\mathrm{t}\mathrm{r}\mathrm{e}\mathrm{a}\mathrm{t}\mathrm{e}\mathrm{d}\:}}\right]\times\:100$$

#### Time-kill kinetics assay

The bactericidal activity of the tested formulations was investigated using the macro-broth dilution method to track time-dependent microbial reduction^26^. Standardized suspensions (~ 2 × 10⁵ CFU/mL) were exposed to the tested formulations and incubated at 37 °C with continuous shaking. Aliquots were withdrawn at predetermined intervals, serially diluted, plated onto Luria–Bertani (LB) agar, and incubated for 24 h. Colony counts (CFU/mL) were recorded, and the reduction percentage was calculated using Eq. (5). Monitoring up to 96 h allowed evaluation of both the rate and sustainability of microbial killing^27^. Furthermore, the complete eradication time for biofilm-forming pathogens was determined to quantitatively assess the formulations’ antimicrobial potency.

5 $$\:\mathrm{P}\mathrm{e}\mathrm{r}\mathrm{c}\mathrm{e}\mathrm{n}\mathrm{t}\mathrm{a}\mathrm{g}\mathrm{e}\:\mathrm{o}\mathrm{f}\:\mathrm{r}\mathrm{e}\mathrm{d}\mathrm{u}\mathrm{c}\mathrm{t}\mathrm{i}\mathrm{o}\mathrm{n}\:\mathrm{i}\mathrm{n}\:\mathrm{g}\mathrm{r}\mathrm{o}\mathrm{w}\mathrm{t}\mathrm{h}=\left[\frac{\left({\mathrm{log}}_{10}{\mathrm{C}\mathrm{o}\mathrm{l}\mathrm{o}\mathrm{n}\mathrm{i}\mathrm{e}\mathrm{s}\:\mathrm{c}\mathrm{o}\mathrm{u}\mathrm{n}\mathrm{t}}_{\mathrm{U}\mathrm{n}\mathrm{t}\mathrm{r}\mathrm{e}\mathrm{a}\mathrm{t}\mathrm{e}\mathrm{d}}-{\mathrm{log}}_{10}{\mathrm{C}\mathrm{o}\mathrm{l}\mathrm{o}\mathrm{n}\mathrm{i}\mathrm{e}\mathrm{s}\:\mathrm{c}\mathrm{o}\mathrm{u}\mathrm{n}\mathrm{t}}_{\mathrm{T}\mathrm{r}\mathrm{e}\mathrm{a}\mathrm{t}\mathrm{e}\mathrm{d}}\right)}{{\mathrm{log}}_{10}{\mathrm{C}\mathrm{o}\mathrm{l}\mathrm{o}\mathrm{n}\mathrm{i}\mathrm{e}\mathrm{s}\:\mathrm{c}\mathrm{o}\mathrm{u}\mathrm{n}\mathrm{t}}_{\mathrm{U}\mathrm{n}\mathrm{t}\mathrm{r}\mathrm{e}\mathrm{a}\mathrm{t}\mathrm{e}\mathrm{d}}}\right]\times\:100$$

#### Minimal Inhibitory Concentration (MIC), Minimum Bactericidal Concentration (MBC), and Fungicidal Concentration (MFC) determination

The most active composite (dH2) was further assessed for MIC, MBC, and MFC using broth microdilution (1.95–500 µg/mL). Ketoconazole, fluconazole, tetracycline, and ampicillin served as positive controls. MIC values were defined as the lowest concentrations producing ≥ 90% growth inhibition after 24–72 h. Wells exhibiting no visible growth were subculture onto LB agar; ≥99.9% reduction in viability was considered bactericidal or fungicidal. All measurements were conducted in triplicate to ensure reproducibility^28^.

### Cytotoxicity study

The cytocompatibility of the composites was assessed using the MTT assay on Vero cells and MG-63 osteoblast-like cells. Cells were seeded at 1 × 10^5^ cells/well in 96-well plates and incubated overnight for attachment. They were then treated with composite concentrations ranging from 62.5 to 1000 µg/mL for 24 h. Following incubation, MTT solution (20 µL, 5 mg/mL) was added and incubated for an additional 4 h. Formazan crystals were solubilized in DMSO (100 µL), and absorbance was measured at 570 nm. Cell viability was calculated using Eq. (6). The assay enabled evaluation of cytocompatibility in both normal mammalian cells (Vero) and human osteoblast-like cells (MG-63)^29^.

6 $$\:\mathbf{C}\mathbf{e}\mathbf{l}\mathbf{l}\:\mathbf{V}\mathbf{i}\mathbf{a}\mathbf{b}\mathbf{i}\mathbf{l}\mathbf{i}\mathbf{t}\mathbf{y}\:\left(\mathbf{\%}\right)=\left[\frac{\mathrm{A}\mathrm{b}\mathrm{s}\mathrm{o}\mathrm{r}\mathrm{b}\mathrm{a}\mathrm{n}\mathrm{c}\mathrm{e}\:\mathrm{o}\mathrm{f}\:\mathrm{t}\mathrm{r}\mathrm{e}\mathrm{a}\mathrm{t}\mathrm{e}\mathrm{d}\:\mathrm{c}\mathrm{e}\mathrm{l}\mathrm{l}\mathrm{s}}{\mathrm{A}\mathrm{b}\mathrm{s}\mathrm{o}\mathrm{r}\mathrm{b}\mathrm{a}\mathrm{n}\mathrm{c}\mathrm{e}\:\mathrm{o}\mathrm{f}\:\mathrm{c}\mathrm{o}\mathrm{n}\mathrm{t}\mathrm{r}\mathrm{o}\mathrm{l}\:\mathrm{c}\mathrm{e}\mathrm{l}\mathrm{l}\mathrm{s}}\right]\times\:100\:$$

All experiments were performed in triplicate, and data are presented as mean ± standard deviation (M ± SD).

### Statistical analysis

Each experiment was repeated three times, and the outcomes were presented as the mean ± standard deviation (M ± SD). Statistical evaluation was carried out using one-way ANOVA, followed by Tukey’s post hoc analysis, employing Minitab version 18. A p-value of ≤ 0.05 was considered statistically significant.

## Results and discussion

### Mechanistic pathway of B6 synthesis and chitosan modification

The synthesis of 2-ethoxy-4-formylphenyl 2,3-dioxo-1,2,3,4-tetrahydroquinoxaline-6-sulfonate (B6) was accomplished through a well-defined multistep route involving the sequential formation of intermediates B0 and B1. Initially, condensation of *o*-phenylenediamine with oxalic acid under acidic conditions afforded 2,3-(1 H,4 H)-quinoxalinedione (B0) via intramolecular cyclization, establishing the quinoxaline core structure^17^. Subsequent chlorosulfonation of B0 using chlorosulfonic acid selectively introduced a sulfonyl chloride (–SO_2_Cl) group at the 6-position through an electrophilic aromatic substitution mechanism, yielding the activated intermediate B1. This transformation rendered the quinoxaline scaffold highly reactive toward nucleophilic substitution. In the final step, the phenolic hydroxyl group of ethyl vanillin reacted with the sulfonyl chloride moiety of B1 to form a stable sulfonate ester (–SO_2_–O–) linkage, generating the target al.dehyde-functionalized quinoxaline derivative (B6). The presence of a free aldehyde group in B6 enables subsequent covalent coupling with polymeric amines^30^.

**Scheme 1:**
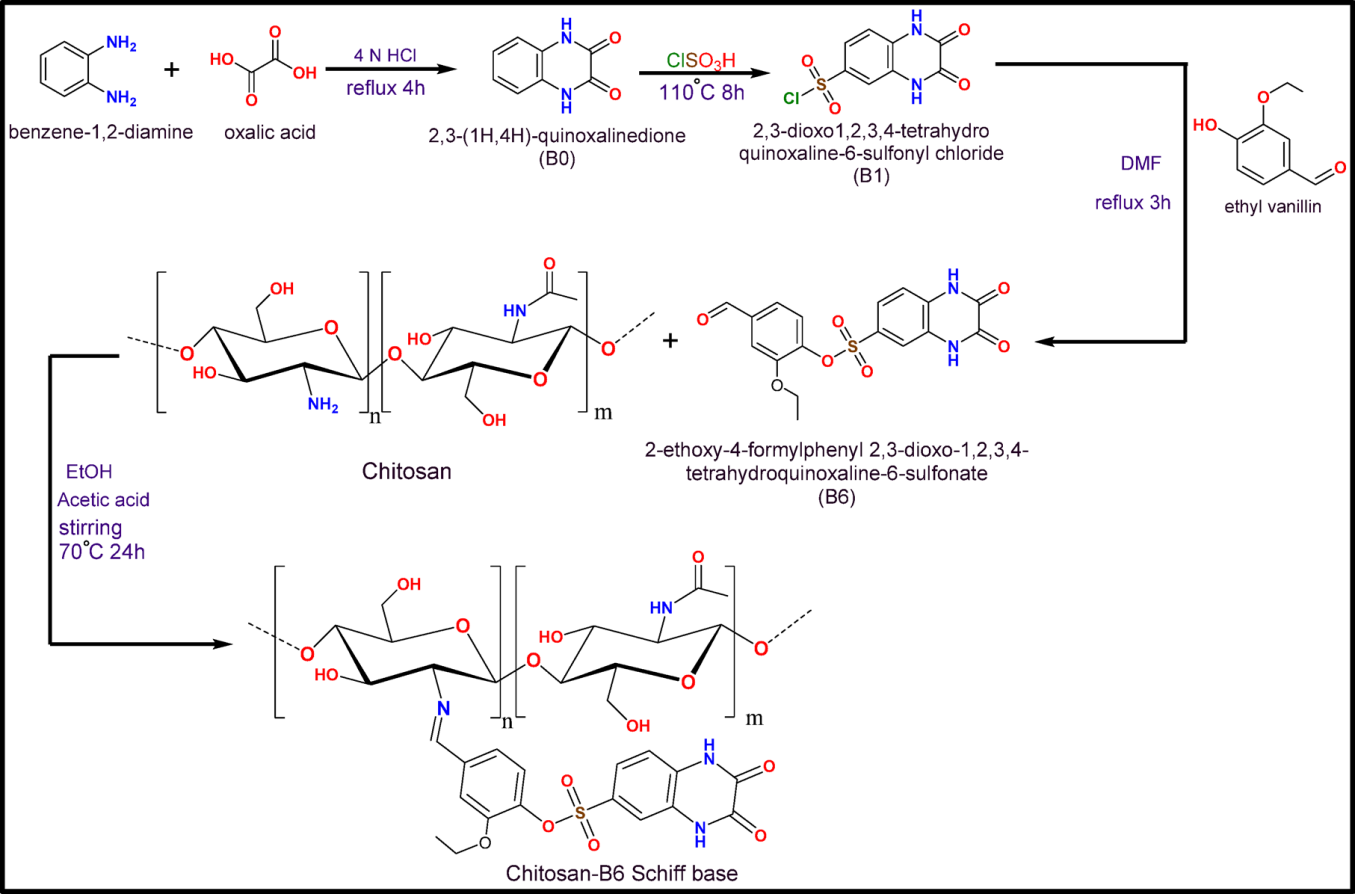
Proposed synthesis route of the chitosan–B6 Schiff base derivative.

Modification of chitosan was achieved through Schiff base condensation between the aldehyde functionality of B6 and the primary amino groups of chitosan under mildly acidic conditions, resulting in the formation of imine (C = N) linkages^31^. Although imine bonds are generally susceptible to hydrolysis under strongly acidic conditions, Schiff base formation under mildly acidic media has been widely reported for chitosan-based systems, where acetic acid is primarily used to solubilize chitosan and facilitate the condensation reaction^12^. The conjugated quinoxaline moiety contributes to partial stabilization of the imine linkage through electron delocalization. In addition, the polymeric chitosan matrix may restrict molecular mobility and limit water accessibility, thereby reducing hydrolytic degradation^14^. Furthermore, the reaction conditions were maintained within a mildly acidic range, which supports imine formation while minimizing bond cleavage. Control over the B6-to-chitosan molar ratio allowed systematic regulation of the degree of substitution, providing a versatile approach to tune the chemical functionality of the polymer backbone. The overall synthetic strategy for B6 and its corresponding chitosan Schiff base derivatives is illustrated in Scheme 1.

### Physicochemical properties

#### Degree of substitution (DS) analysis

The degree of substitution (DS) was determined to quantify the extent of Schiff base formation between chitosan and the quinoxaline aldehyde B6 at different feed ratios. Nitrogen content was measured using the Kjeldahl method, which has been widely validated for evaluating substitution levels in chitosan derivatives^32^. The calculated nitrogen contents and corresponding DS values are summarized in Supplementary Table 1. Unmodified chitosan exhibited a nitrogen content of 6.66%, whereas the chitosan–B6 Schiff base derivatives (d1–d4) showed progressively higher nitrogen contents of 7.45%, 7.30%, 7.10%, and 6.90%, respectively, reflecting the successful incorporation of the nitrogen-containing quinoxaline moiety. Based on these values, the DS was calculated as 0.631 (d1), 0.623 (d2), 0.613 (d3), and 0.602 (d4). The systematic increase in nitrogen content with increasing aldehyde loading confirms the gradual incorporation of B6 and formation of imine linkages (–C = N). This controlled variation in DS demonstrates efficient Schiff base formation and provides a quantitative basis for correlating chemical modification with subsequent changes in composite structure and biological performance.

#### Ion exchange capacity (IEC)

The ion exchange capacity (IEC) was evaluated to assess the extent of chemical modification of chitosan and the availability of residual free amino groups after Schiff base formation. IEC values were calculated according to Eq. (2), based on acid–base back-titration. Native chitosan exhibited the highest IEC value (12.24 meq/g), reflecting the abundance of free –NH_2_ groups along the polymer backbone. Upon functionalization with the quinoxaline aldehyde (B6), a systematic decrease in IEC was observed, following the order d4 (11.31) > d3 (9.12) > d2 (6.24) > d1 (3.13 meq/g)^33^. This progressive decline in IEC directly reflects the consumption of primary amine groups through imine (C = N) formation during Schiff base condensation, thereby reducing the number of ion-exchangeable sites. Notably, the nitrogen atoms within the quinoxaline ring do not contribute to IEC under the acidic titration conditions (0.1 M H_2_SO_4_), as they are involved in conjugated heterocyclic systems and are not readily protonated. The close correlation between IEC values and the degree of substitution (DS) confirms the effectiveness of Schiff base formation and highlights how controlled chemical modification governs the ion-exchange behavior of chitosan.

#### Solubility test

The solubility behavior of chitosan and its Schiff base derivatives (d1–d4) in various solvents is summarized in Table 3. Native chitosan was insoluble in water due to extensive intermolecular hydrogen bonding but readily dissolved in 1% acetic acid as a result of amino group protonation. In polar aprotic solvents such as DMSO, chitosan exhibited swelling rather than complete dissolution, consistent with previously reported behavior^34^. Schiff base formation markedly altered the solubility profile of chitosan in a manner dependent on the degree of substitution and IEC. Highly substituted derivatives (d1 and d2) became insoluble in 1% acetic acid, reflecting the reduced availability of free amino groups. In contrast, lower-substituted samples (d3 and d4) retained partial solubility or swelling behavior.

In organic solvents, d1 showed complete dissolution in DMSO, whereas the remaining derivatives exhibited swelling in DMF, indicating that imine density plays a key role in governing solvent–polymer interactions. These findings provide further indirect evidence of successful Schiff base formation and demonstrate how controlled chemical modification enables systematic tuning of chitosan solubility characteristics. For NMR measurements, the limited solubility of some derivatives in DMSO was overcome by adding a small amount of TFA-d to the DMSO-d6 solvent system. Therefore, the NMR conditions do not contradict the solubility results reported in Table 3, since the latter were evaluated using pure solvents under standardized conditions.

**Table 3:** Solubility behavior of chitosan and Schiff base derivatives (d1–d4) in various solvents.

Samples	Distilled water	THF	Acetone	Methanol	1% acetic acid	DMSO	DMF
Chitosan	-	-	-	-	+	±	-
d1	-	-	-	-	-	+	±
d2	-	-	-	-	-	±	±
d3	-	-	-	-	±	±	±
d4	-	-	-	-	±	±	-

(+ soluble, - insoluble, ± swell).

### Instrumental analysis

#### FT-IR analysis

**Fig. 1:**
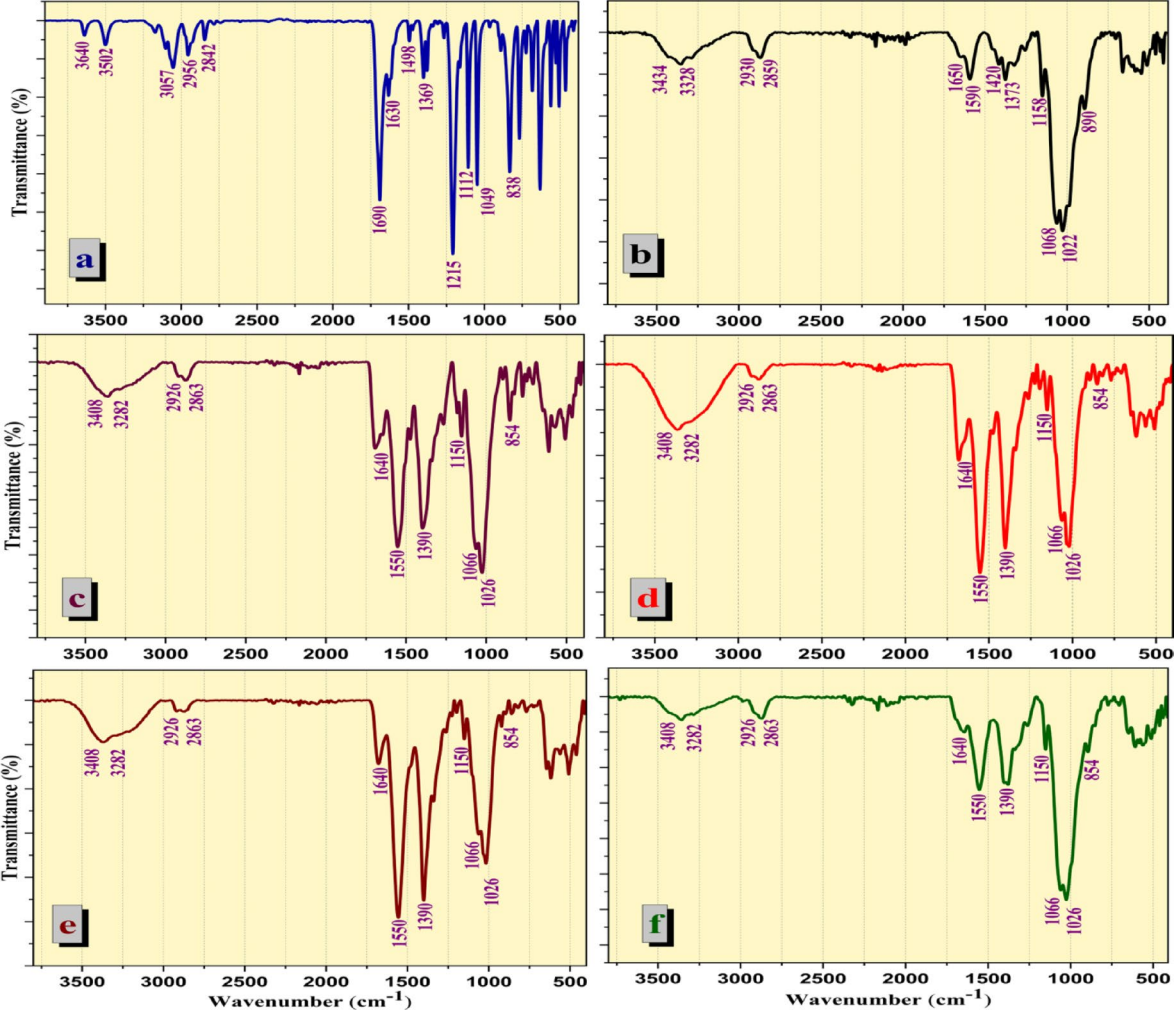
FTIR spectra of (**a**) B6, (**b**) native chitosan, and Schiff base derivatives: (**c**) d1, (**d**) d2, (**e**) d3, and (**f**) d4.

FT-IR spectroscopy was used to monitor functional-group changes during synthesis of B6, native chitosan and the resultant Schiff-base conjugates (Fig. 1). The spectrum of B6 displayed diagnostic absorptions attributable to the quinoxaline and ethoxy-vanillin moieties. Strong bands at 3640 and 3502 cm^− 1^ (assigned to N–H or strongly hydrogen-bonded N–H/–OH stretches), aromatic C–H stretching near 3057 cm^− 1^, and aliphatic C–H stretches of the ethoxy group at 2956 and 2842 cm^− 1^. The aldehydic C = O appears as a sharp feature at 1690 cm^− 1^, whereas the quinoxaline amide-type C = O is observed near 1630 cm^− 1^. Characteristic sulfonate absorptions are evident at 1369 and 1112 cm^− 1^. Additional bands at 1498, 1215, 1049, and 838 cm^− 1^ are consistent with aromatic C = C, C–N, C–O and out-of-plane aromatic C–H modes respectively^35^.

Native chitosan exhibited the expected spectral signatures: a broad O–H/N–H envelope between 3500 and 3100 cm^− 1^ with discrete contributions at 3434 cm^− 1^ (O–H) and 3328 cm^− 1^ (N–H), weak aliphatic C–H bands at 2930 and 2859 cm^− 1^, an amide I (residual N-acetyl) near 1652 cm^− 1^, N–H bending at 1590 cm^− 1^, CH_2_/CH_3_ bending at 1420 and 1373 cm^− 1^, asymmetric C–O–C at 1158 cm^− 1^, C–O stretches at 1068 and 1022 cm^− 1^, and the monosaccharide ring out-of-plane mode at 890 cm^− 1^^36^.

Following Schiff base formation, the broad band at 3100–3500 cm^− 1^ weakened, with the N–H peak at 3328 cm^− 1^ disappearing and the O–H peak shifting from 3434 to 3408 cm^− 1^, indicating altered hydrogen bonding. The aldehyde C = O peak at 1690 cm^− 1^ also disappeared, confirming imine formation. New peaks appeared at 3282, 2926, and 2863 cm^− 1^, corresponding to amide N–H, aromatic C–H (quinoxaline), and aliphatic C–H (chitosan), respectively. Bands at 1150, 1066, and 1026 cm^− 1^ were attributed to asymmetric C–O–C stretching and C–O stretching, while the 854 cm^− 1^ peak was linked to aromatic C–H bending of the quinoxaline moiety^37^.

A strong new band at 1550 cm^− 1^, absent in chitosan and B6, confirmed C = N stretching and successful imine linkage formation. Its intensity increased with the degree of substitution (DS), being strongest in d1–d2 and weaker in d4. A shoulder at 1640 cm^− 1^, intermediate between B6 (1630 cm^− 1^) and chitosan (1650 cm^− 1^), reflected overlapping C = O and C = N vibrations^38^. A new band at 1390 cm^− 1^, absent in native chitosan, is attributed to contributions from the sulfonate (SO_2_) group of B6 and modifications in the chitosan backbone after Schiff base formation. This band appears in a region close to the characteristic vibrations of the sulfonate group in B6 (1369 cm^− 1^) and the C–N stretching in chitosan (1373 cm^− 1^), suggesting overlapping contributions rather than a true band shift. Collectively, these spectral changes verify the successful grafting of B6 onto chitosan and the formation of the quinoxaline-based Schiff base derivative.

Supplementary Fig. 1 shows the FT-IR spectra of the chitosan–Schiff base/hydroxyapatite composites, confirming the successful incorporation of hydroxyapatite (HAp) into the polymeric matrix. Characteristic phosphate vibrations dominate the 470–1100 cm^− 1^ region. The band at ~ 470 cm^− 1^ corresponds to PO_4_^−3^ bending, while the absorptions at ~ 560 and 601 cm^− 1^ arise from ν_4_ asymmetric bending modes. The strong band near 1020 cm^− 1^, accompanied by a shoulder at ~ 954 cm^− 1^, is assigned to ν_3_ and ν_1_ phosphate stretching vibrations, respectively, indicating the formation of a well-crystallized apatite phase typical of stoichiometric hydroxyapatite^39^.

The weak bands at 1450 cm^− 1^ and 1410 cm^− 1^ are attributed to carbonate (CO_3_^2−^) groups, which are commonly present in trace amounts in hydroxyapatite. These bands suggest the formation of carbonated hydroxyapatite, which is known to enhance the biocompatibility and bioactivity of the material^40^. The very weak band at 1650 cm^− 1^ is likely related to residual water molecules adsorbed on the hydroxyapatite surface or to C = O stretching vibrations from minor impurities or characteristic groups in the Schiff base.

In the composite spectra, the characteristic bands of the chitosan–Schiff base become less distinct due to overlap with the intense phosphate absorptions of HAp. The reduced intensity of the broad O–H/N–H stretching band indicates strong interfacial interactions, likely hydrogen bonding and electrostatic attractions, between the polymer functional groups and the hydroxylated HAp surface. This spectral attenuation supports homogeneous dispersion and intimate coupling of HAp within the Schiff-base-modified chitosan network, confirming the formation of a structurally integrated organic–inorganic composite.

#### ^1^H NMR and ^13^C NMR analysis

**Fig. 2:**
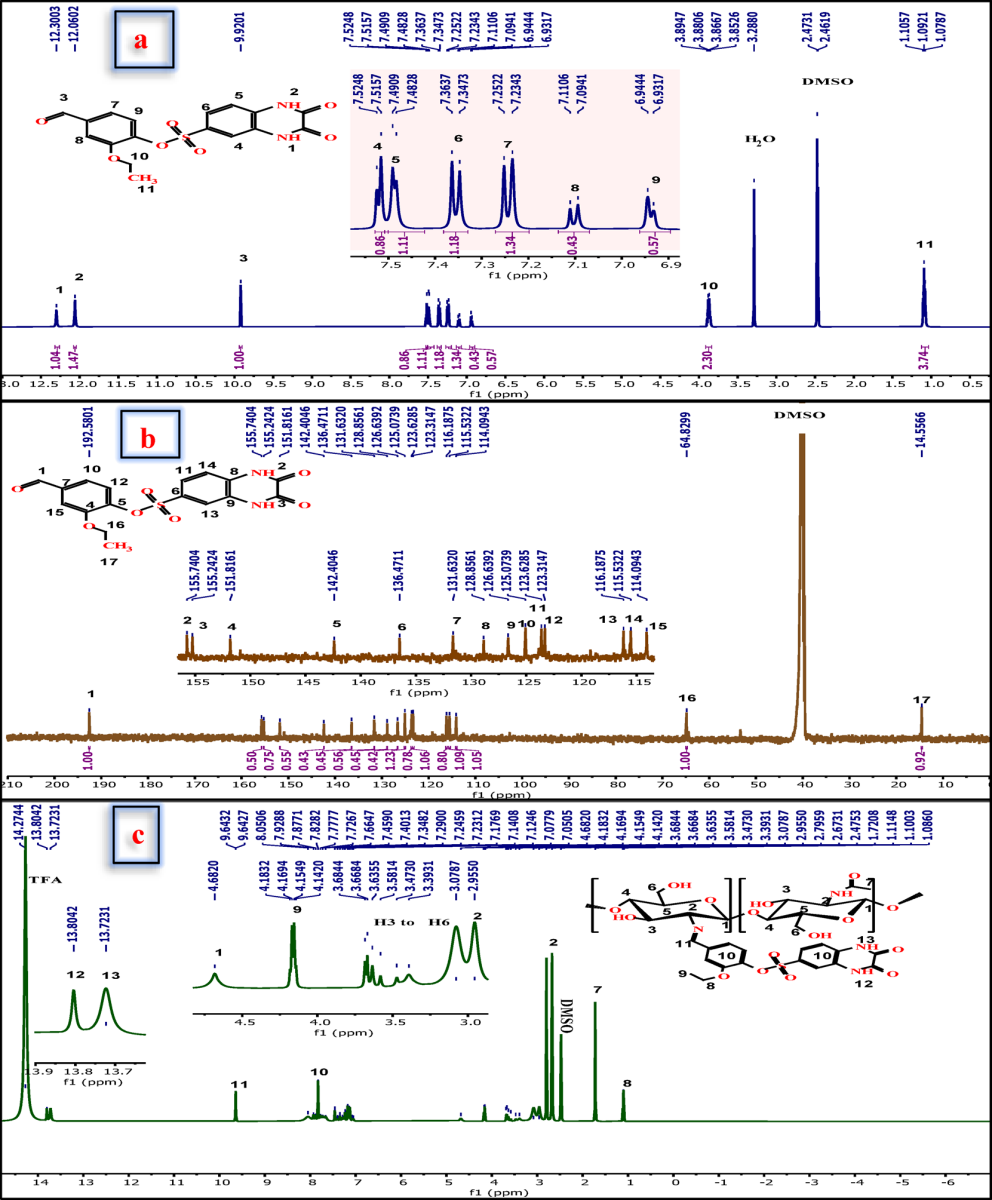
(**a**) ^1^H NMR spectrum of B6 in DMSO-*d*_6_, (**b**) ^13^C NMR spectrum of B6 in DMSO-*d*_6_, and (**c**) ^1^H NMR spectrum of chitosan-B6 Schiff base in a mixture of TFA-*d* and DMSO-*d*_6_.

To confirm the successful synthesis of quinoxaline aldehyde derivative (B6), ^1^H NMR (500 MHz, DMSO-d_6_) was performed, and the obtained spectrum is shown in Fig. 2a. Singlet peaks at 12.30 ppm and 12.06 ppm correspond to the two amide (-NH) protons of the quinoxaline-dione moiety^41^. The aldehydic proton appears as a singlet at δ 9.92 ppm, confirming the formyl functionality. Aromatic resonances were observed between δ 6.93–7.52 ppm, assignable to protons on both quinoxaline and ethyl-vanillin rings. H_4_ (δ 7.52 ppm) exhibited meta-coupling, while H_5_ and H_6_ appeared as doublets at δ 7.49 ppm and 7.36 ppm. The ethyl-vanillin ring displayed doublets at δ 7.24, 7.10, and 6.94 ppm (H_7_-H_9_). The methylene and methyl protons of the ethoxy group resonated as a quartet at δ 3.87 ppm and a triplet at δ 1.09 ppm, respectively, confirming the ethoxy substituent.

The ^13^C NMR (126 MHz, DMSO-d_6_) spectrum of B6 (Fig. 2b) provided further confirmation of its molecular structure, with clear assignments for all carbon signals. The aldehyde carbon (C1) appeared at δ 192.58 ppm, and the amide carbonyls (C2 and C3) at δ 155.74 and 155.24 ppm, confirming the formyl group and the quinoxaline-dione core. The oxygenated carbons within the structure displayed characteristic shifts: C4 and C5, linked to the ethoxy and sulfonate groups, resonated at δ 151.81 and 142.40 ppm, respectively. Additionally, C6, the carbon bonded directly to the sulfur atom within the sulfonate group, gave a signal at δ 136.47 ppm and the formyl-substituted carbon C7, adjacent to the formyl group, appeared at δ 131.63 ppm. Meanwhile, the amide-linked carbons (C8 and C9) displayed signals at δ 128.85 ppm and 126.63 ppm, respectively and C10 carbon, *ortho* to the formyl group, appeared at δ 125.07 ppm. Similarly, C11 and C12, both ortho to the sulfonate group in the quinoxaline and ethyl vanillin rings, were observed at δ 123.62 ppm and 123.31 ppm, respectively. The remaining aromatic carbons of the quinoxaline ring, C13 and C14, were identified at δ 116.18 ppm and 115.53 ppm, and C15, between the formyl and ethoxy groups, resonated at δ 114.09 ppm. The ethyl chain exhibited characteristic aliphatic signals at δ 64.82 ppm (–CH_2_–) and δ 14.56 ppm (–CH_3_). These data confirm the expected molecular architecture of B6 and the successful incorporation of the sulfonate and aldehyde functionalities.

The ¹H NMR spectrum of the chitosan–B6 Schiff base (Fig. 2c) exhibited resonances characteristic of both chitosan and B6. The anomeric proton (H_1_) appeared at δ 4.68 ppm, while (H_2_) resonated near δ 2.95 ppm with additional minor signals at 2.67 and 2.79 ppm, reflecting heterogeneous local environments. The multiple region between δ 3.07–3.68 ppm corresponded to ring protons (H_3_–H_6_), and the residual acetyl methyl (H_7_) appeared as a singlet at δ 1.72 ppm^42^. Within the ethoxy substituent, the terminal methyl protons (H_8_) appeared as a triplet at 1.10 ppm, while the adjacent methylene protons (H_9_) showed a quartet at 4.16 ppm. Aromatic protons of the quinoxaline unit resonated downfield at δ 7.12–7.92 ppm. The disappearance of the aldehydic signal (δ 9.92 ppm) and emergence of a new singlet at δ 9.64 ppm, attributed to the imine proton (H_11_), confirm Schiff base formation. Two singlets at δ 13.80 and 13.72 ppm correspond to the amide protons (H_12_, H_13_) of the quinoxaline moiety, slightly downfield due to intramolecular hydrogen bonding. Together, the ¹H and ¹³C NMR data unequivocally support the formation of the new quinoxaline aldehyde (B6) and its subsequent incorporation into the chitosan matrix through imine linkage.

#### MS spectrometry

**Fig. 3:**
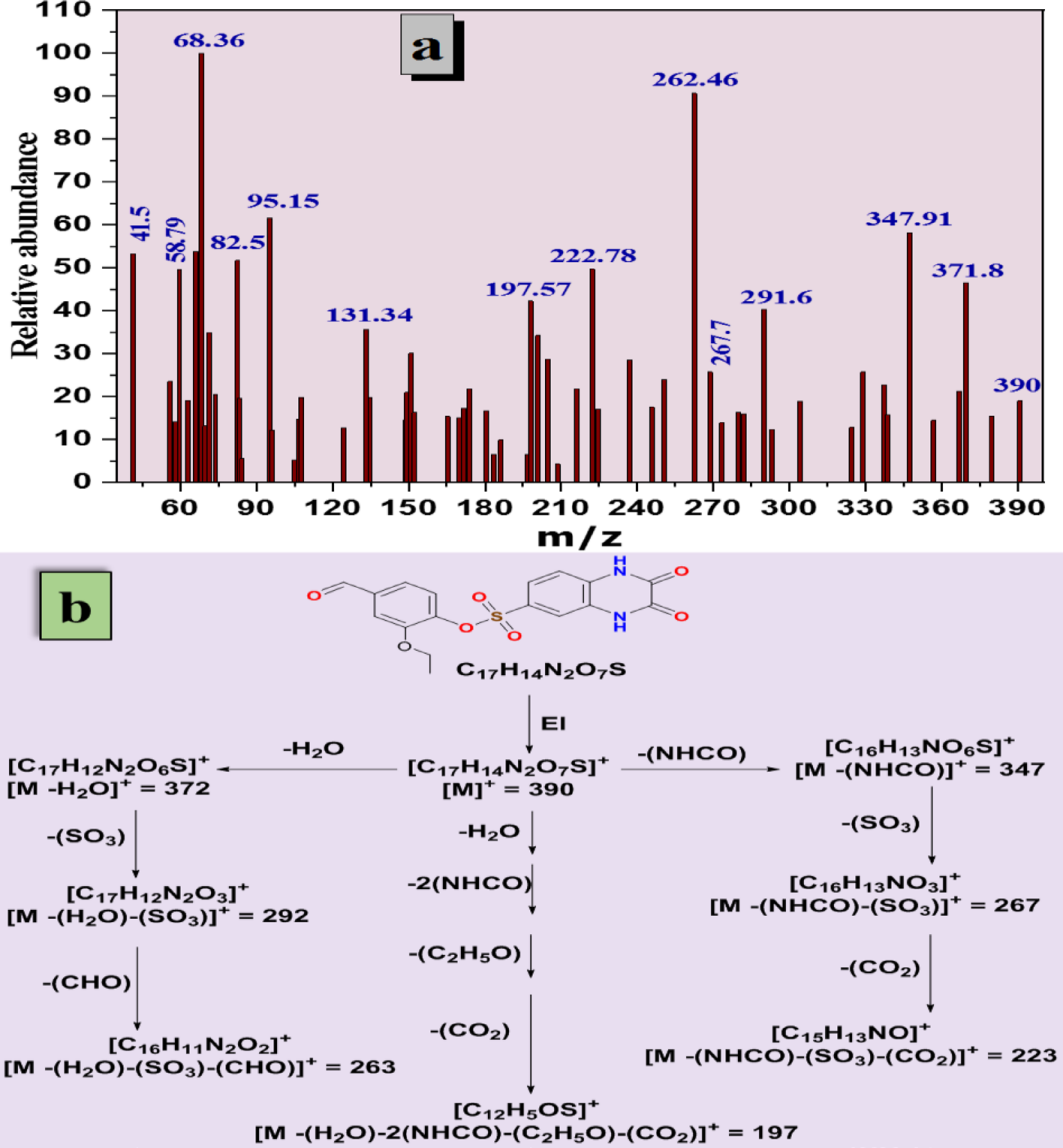
(**a**) Mass spectrum of the quinoxaline aldehyde derivative(B6). (**b**) Proposed fragmentation pathway highlighting key fragment ions.

Mass spectrometry of the synthesized quinoxaline compound B6 showed a molecular ion peak at *m/z* 390, consistent with the calculated mass of [C_17_H_14_N_2_O_7_S]^+^, thus validating the proposed molecular structure (Fig. 3a). The observed fragmentation profile further supports the structural integrity of B6, following expected neutral losses (Fig. 3b).

The primary fragmentation pathway involves the loss of an NHCO moiety, leading to a fragment at *m/z* 347 [C_16_H_13_NO_6_S]^+^. Subsequent elimination of SO₃ results in a peak at *m/z* 267 [C_16_H_13_NO_3_]^+^, followed by the loss of CO₂, yielding a fragment at *m/z* 223 [C_15_H_13_NO]^+^.

An alternative fragmentation route includes the initial loss of H₂O, forming a fragment at *m/z* 372 [C_17_H_12_N_2_O_6_S]^+^, followed by the elimination of SO₃ to generate the peak at *m/z* 292 [C_17_H_12_N_2_O_3_]^+^. Further loss of a CHO moiety results in the *m/z* 263 [C_16_H_11_N_2_O_2_]^+^ ion.

A deeper fragmentation pathway involves multiple consecutive neutral losses, where H₂O, two NHCO groups, an ethoxy (-C₂H₅O) moiety, and CO₂ are eliminated, leading to a significant fragment at *m/z* 197 [C_12_H_5_OS]^+^.

The base peak was observed at *m/z* 68, suggesting the formation of a highly stable ion. Based on its mass and possible structural rearrangement, this fragment is best assigned to the [C_4_H_6_N]^+^ ion, which could arise from a resonance-stabilized imine or pyrrolyl-like structure. The stability of this ion is likely due to delocalization of the positive charge across the conjugated system, making it the most abundant fragment in the spectrum^43^.

Additionally, smaller fragment ions observed in the spectrum include *m/z* 131 ([C_8_H_5_NO]^+^, likely a quinoxaline derivatives radical cation), *m/z* 95 ([C_6_H_7_O]⁺, corresponding to a hydroxybenzyl cation), *m/z* 82 ([C_5_H_6_O]^+^, suggestive of a phenolic derivative cation), *m/z* 58 ([C_2_H_4_NO]^+^, possibly an acetimidoyl or oxaziridinium ion), and *m/z* 41 (C_3_H_5_, indicative of an allyl or propargyl cation), all of which correspond to structurally stable moieties derived from core skeleton cleavage^44^. Altogether, the observed fragmentation pattern aligns well with the proposed structure and confirms the successful synthesis of the B6 molecule.

#### XRD analysis

**Fig. 4:**
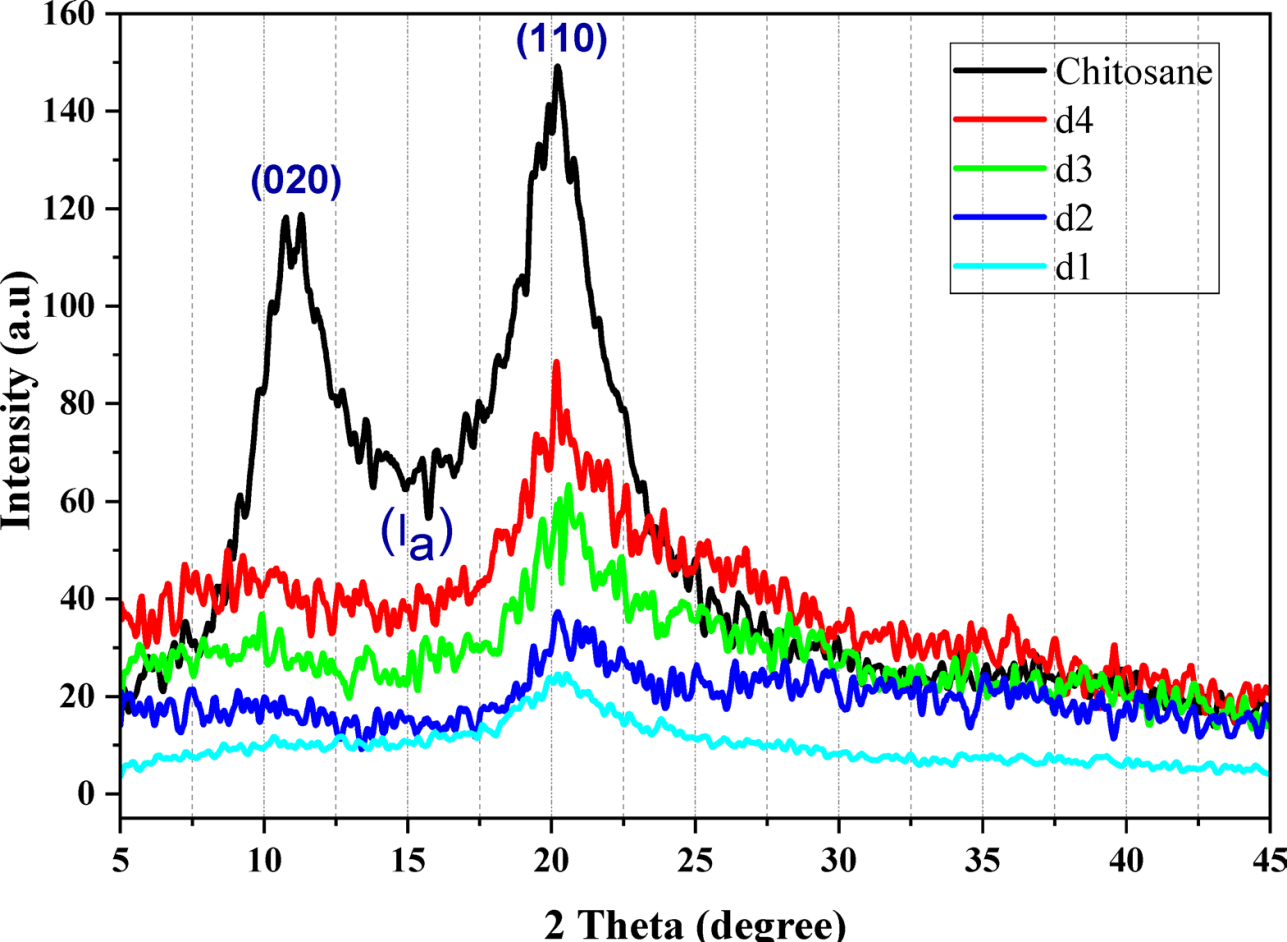
Stacked XRD patterns of chitosan and Schiff base derivatives (d1–d4), with each sample indicated by a distinct color.

Figure 4 shows XRD patterns of native chitosan and its B6 Schiff base derivatives. Peaks at 2θ = 11.32° and 20° in unmodified chitosan, corresponding to the 020 and 110 planes, confirm its semi-crystalline nature. The broad diffraction at 11.32° (*d* = 7.87 Å) is associated with the hydrated polymorphic “tendon” form of chitosan^45^. After Schiff base formation with B6, the (020) reflection disappeared completely, while the (110) peak at 20° decreased in intensity and slightly shifted to higher 2θ values. This shift, with a decrease in d-spacing from 4.42 Å to 4.27 Å, indicates lattice contraction and disruption of the intermolecular hydrogen-bonded network. These structural modifications confirm that the introduction of imine linkages disturbs the regular packing of chitosan chains^46^. The calculated crystallinity index (CI), summarized in Supplementary Table 2, decreased from 67% for native chitosan to 59.5%, 56.7%, 53%, and 48.5% for derivatives d4, d3, d2, and d1, respectively. This progressive reduction reflects the increasing substitution of amino groups by imine bonds, which lowers chain ordering and enhances amorphous characteristics^47^. The decline in crystallinity suggests that Schiff base formation disrupts molecular alignment, improving solubility, chemical reactivity, and interfacial compatibility with hydroxyapatite in subsequent composite systems.

**Fig. 5:**
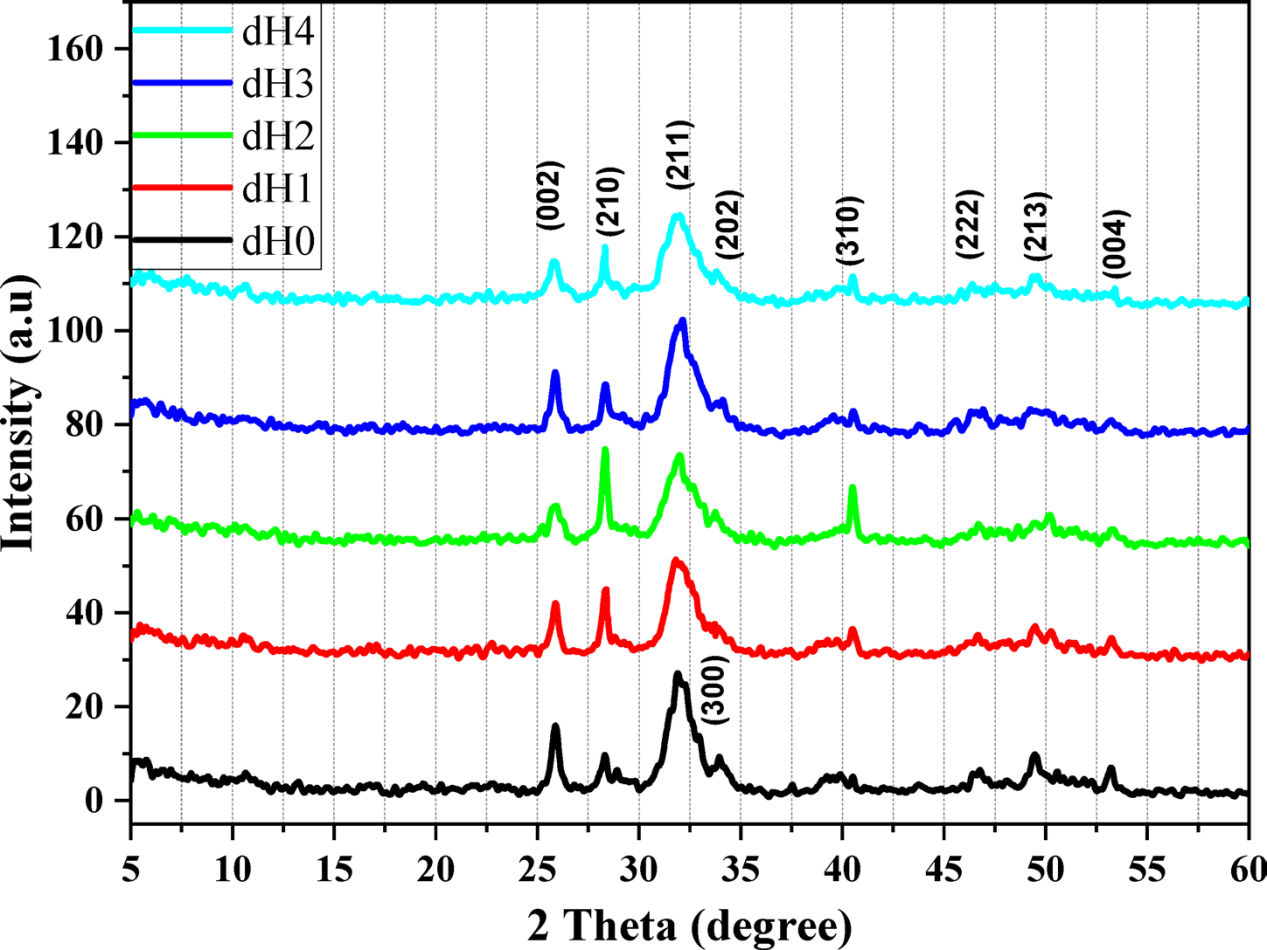
Stacked XRD patterns of hydroxyapatite composites (dH0–dH4), with each sample shown in a distinct color.

Figure 5 presents the XRD patterns of hydroxyapatite (HAp) composites containing unmodified chitosan (dH0) and Schiff base derivatives (dH1–dH4). The analysis evaluates the crystal structure of HAp and the influence of chitosan modification on phase composition and crystallinity. Quantitative parameters, including FWHM values (from Gaussian peak fitting in OriginPro) and crystallite sizes (calculated using Scherrer’s equation), are summarized in Supplementary Table 3, providing insight into the crystallite evolution within the composites. All samples showed HAp diffraction peaks at 25.8° (002), 28.3° (210), 31.9°–32.1° (211), 33.8°–34.0° (202), 39.5°–40.5° (310), 46.6°–46.9° (222), 49.5°–49.9° (213), and 53.2°–53.4° (004), matching with (JCPDS card No. 09-0432 / 01-077-8798)^48^. These peaks confirm the preservation of HAp hexagonal P6₃/m phase within the composites. A notable feature was the disappearance of the (300) reflection at 33.1°, detected only in dH0, suggesting that Schiff base modification alters the HAp lattice through interfacial interactions between surface phosphate groups and Schiff base functional moieties. Moreover, the principal reflections ((002), (210), (211), (202)) in dH1–dH4 shifted slightly toward higher 2θ values, reflecting decreased d-spacing and indicating lattice contraction due to molecular-level interactions^49^. Furthermore, the refined crystallographic parameters obtained from lattice analysis (Supplementary Table 4) confirmed the hexagonal P6_3_/m structure for all composites. A noticeable lattice contraction was observed for the dH2 composite (a = 3.6290 Å, c = 5.8900 Å, V = 67.18 Å³), suggesting a stronger interaction between Schiff base functional groups and the HAp lattice at this composition. This structural distortion is consistent with the observed peak shifts and further confirms that Schiff base incorporation induces localized molecular interactions without compromising the integrity of the apatite lattice. Crystallite size increased from 9.97 nm (dH0) to 18.21 nm (dH1), indicating that Schiff base incorporation facilitated crystal growth by promoting nucleation. As Schiff base content decreased (dH2–dH4), crystallite size declined (12.90–10.89 nm), indicating reduced steric hindrance enabled more controlled crystallization.

#### XPS analysis

X-ray photoelectron spectroscopy (XPS) was employed to provide direct evidence of chemical interactions within the synthesized chitosan Schiff base and its hydroxyapatite-based composites. High-resolution spectra of C1s, N1s, O1s, P2p, Ca2p, and S2p were recorded for the representative samples d2 (chitosan–B6 Schiff base), dH0 (chitosan/HAp), and dH2 (Schiff base–modified chitosan/HAp). The corresponding binding energies and peak assignments are summarized in Supplementary Table 5.

The C1s spectrum of sample d2 (Supplementary Fig. 2a) exhibits three main components centered at ~ 285.37 eV, ~ 286.74 eV, and ~ 287.95 eV, attributed to C–C/C–H, C–O or C–N, and C = N/C = O environments, respectively. The appearance of the high-binding-energy component at ~ 287.95 eV provides clear evidence for imine (C = N) formation, confirming successful Schiff base condensation between chitosan amino groups and the aldehyde functionality of B6^50^. For the HAp-containing composites (dH0 and dH2), the C1s spectra (Supplementary Fig. 2b,c) were dominated by overlapping carbon contributions arising from the polymer backbone and surface-adsorbed carbon species, resulting in reduced resolution of individual carbon functionalities. In particular, the imine-related contribution in the C1s spectrum of dH2 becomes less distinguishable due to partial surface coverage by the inorganic hydroxyapatite phase and overlap with C–O/C–N environments.

The N1s spectrum of d2 (Supplementary Fig. 3a) is composed of three contributions at ~ 397.28 eV, ~ 400.19 eV, and ~ 402.99 eV, corresponding to imine nitrogen (C = N), free amine nitrogen (–NH₂), and protonated nitrogen species, respectively. The presence of a distinct low-binding-energy imine peak at ~ 397.28 eV provides strong and direct evidence for Schiff base formation. For dH0 (Supplementary Fig. 3b), only amine-type nitrogen is observed (~ 400.01–400.55 eV), consistent with native chitosan. In contrast, dH2 (Supplementary Fig. 3c) exhibits both amine (~ 400.0 eV) and imine (~ 401.49 eV) components, confirming that the Schiff base structure remains chemically intact after HAp deposition^51^.

The O1s spectrum of d2 (Supplementary Fig. 4a) consisted of two main components centered at ~ 531.63 and ~ 532.60 eV, attributed to carbonyl, sulfonyl, and ether oxygen species from the quinoxaline Schiff base and chitosan backbone. In the dH0 composite, the O1s region was dominated by a single peak at ~ 531.86 eV, characteristic of phosphate (PO_4_^3−^) oxygen in hydroxyapatite, confirming the formation of a typical apatite lattice. Notably, the O1s spectrum of dH2 revealed an additional low-binding-energy component at ~ 529.32 eV (Supplementary Fig. 4b,c), which can be associated with Ca–O interactions within a more strongly coordinated apatite environment. This additional O1s contribution suggests stronger interfacial interactions between the Schiff base–modified chitosan and the apatite phase, consistent with enhanced organic–inorganic coupling in dH2^52^.

As shown in Supplementary Fig. 5, the Ca2p spectra of both dH0 and dH2 exhibit the characteristic Ca2p_3_/_2_ and Ca2p_1_/_2_ doublet centered at approximately 345.8–345.9 eV and 349.5–349.6 eV, respectively. These binding energies are in excellent agreement with reported values for Ca^2+^ in stoichiometric hydroxyapatite, indicating that the calcium environment remains chemically stable regardless of polymer modification. Importantly, no additional calcium-related components or peak shifts were detected in dH2, confirming that incorporation of the quinoxaline-based chitosan Schiff base does not disrupt the Ca–O coordination framework of the apatite lattice. Similarly, the P2p spectra of dH0 and dH2 (Supplementary Fig. 6) display well-defined doublets at approximately 130.7–130.8 eV (P2p_3_/_2_) and 131.5–131.6 eV (P2p_1_/_2_), corresponding to phosphate (PO_4_^3−^) groups typical of crystalline hydroxyapatite. The close overlap of P2p binding energies between the two composites suggests that the phosphate chemical environment is preserved after Schiff base functionalization. Notably, the absence of peak broadening or additional phosphorus species indicates that no secondary calcium phosphate phases were formed during composite preparation^53^.

Sulfur chemical states were examined to further support the successful incorporation of the sulfonated quinoxaline moiety within the chitosan Schiff base and its hydroxyapatite composite. The S2p signal was clearly detected in samples d2 and dH2, while no sulfur contribution was observed for dH0, consistent with the absence of sulfonated groups in native chitosan/HAp. As summarized in Supplementary Table 5, the S2p binding energies in d2 (~ 163–165 eV) are characteristic of sulfonated aromatic sulfur.

#### TGA measurements

**Fig. 6:**
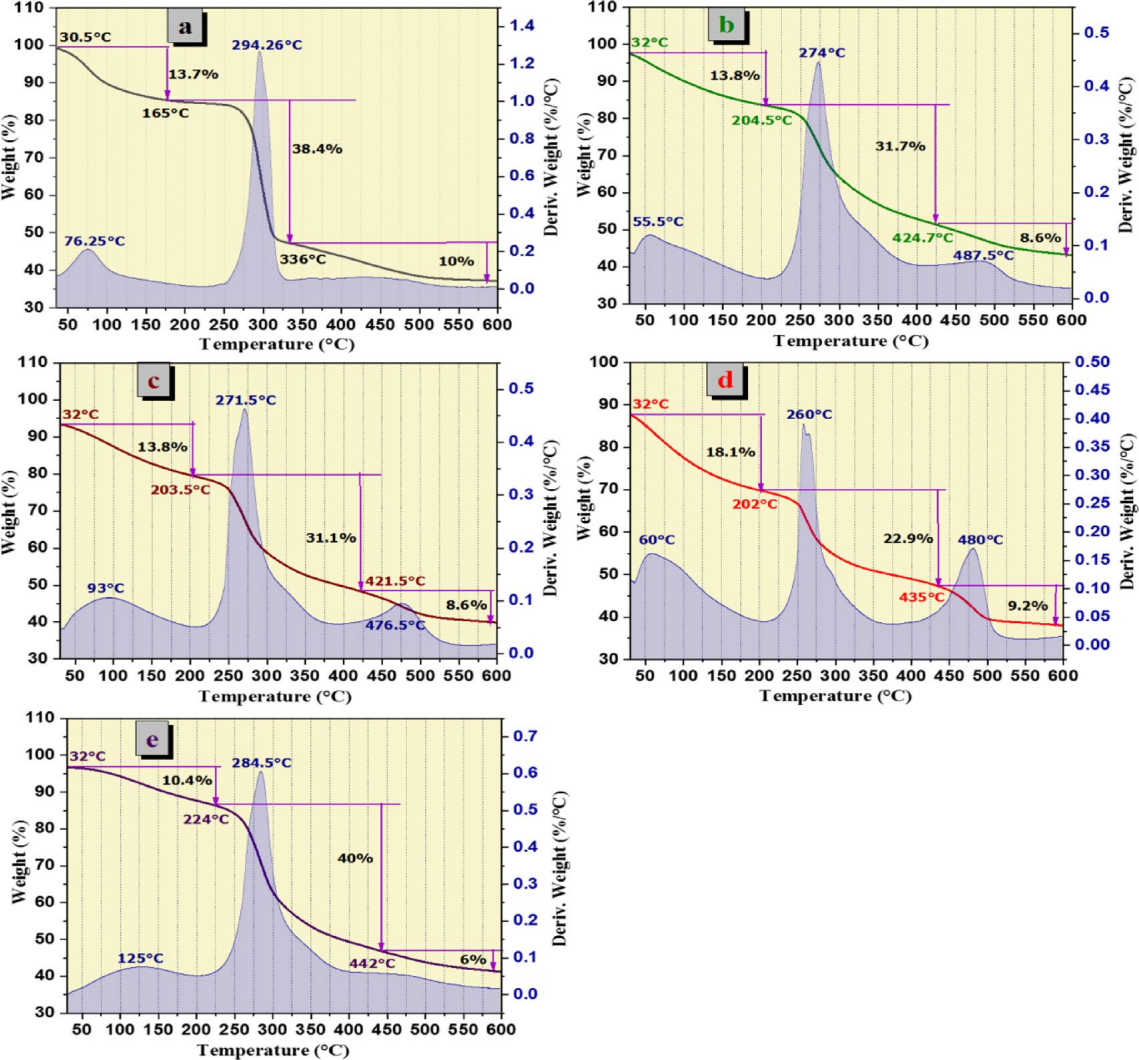
TGA and DTG thermograms of (**a**) chitosan, (**b**) d1, (**c**) d2, (**d**) d3, and (**e**) d4 samples, illustrating thermal degradation behavior.

The thermal degradation behavior of chitosan and its Schiff base derivatives (d1–d4) was investigated by TGA–DTG under nitrogen (Fig. 6), revealing a clear three-step decomposition profile.

The first degradation stage (≈ 30–224 °C) is attributed to the removal of physically adsorbed and bound water. Native chitosan showed a weight loss of ~ 10.4% with a DTG maximum at 76 °C, whereas Schiff base derivatives displayed higher dehydration temperatures (up to 224 °C for d4) and DTG maxima ranging from 55.5 to 125 °C. These variations indicate that Schiff base formation alters the hydrogen-bonding network by consuming free –NH_2_ groups and introducing quinoxaline and sulfonate functionalities, leading to modified water–polymer interactions. In addition, the shift toward higher dehydration temperatures suggests stronger binding of water molecules within the modified polymer network. This behavior is likely due to increased polarity and the presence of sulfonate groups. These functional groups enhance water retention through electrostatic interactions.

The second degradation stage (≈ 165–336 °C) corresponds to saccharide ring dehydration, depolymerization, and cleavage of acetylated and deacetylated units in chitosan. Native chitosan exhibited a sharp DTG maximum at 294 °C, characteristic of rapid, cooperative chain scission. Schiff base derivatives showed broader, less intense peaks at 274 °C, 271.5 °C, 260 °C, and 284.5 °C for d1–d4, indicating a partially disrupted hydrogen-bond network due to bulky substituents and imine formation. Such structural modification generates more heterogeneous local environments, where early degradation can occur near the substituted sites, while the overall polymer matrix remains more thermally resistant. This apparent contradiction—earlier onset of degradation but improved overall thermal resistance—can be explained by the coexistence of two opposing effects. The first is the disruption of crystalline domains due to Schiff base formation, which facilitates the initial degradation. The second is the introduction of rigid aromatic quinoxaline moieties, which enhances thermal stability at higher temperatures. Despite the lower onset temperatures, the total weight loss in this stage (~ 30% for d1–d3, compared to ~ 38% for chitosan) is reduced, confirming that chain scission is more gradual and less extensive. This behavior is consistent with previously reported chitosan Schiff base systems, where partial loss of crystallinity is accompanied by improved thermal resistance due to restricted chain mobility and enhanced intermolecular interactions^54^.

The third degradation stage reflects the decomposition of imine linkages and aromatic substituents introduced through quinoxaline modification. While native chitosan showed only a weak DTG signal above 330 °C, Schiff base derivatives exhibited distinct high-temperature DTG peaks between 442 °C and 487.5 °C. This behavior is attributed to the thermal stability of conjugated aromatic domains and imine (C = N) bonds, which promote char formation and delay complete decomposition. The increased char yield at elevated temperatures further supports the formation of a thermally stable carbonaceous structure, arising from the condensation of aromatic segments and crosslinked imine networks. Consequently, the T_50_ values increased markedly from 310 °C for chitosan to 408–460 °C for the modified derivatives^55^. Notably, derivatives with higher degrees of substitution exhibited more pronounced high-temperature stability, indicating that the density of quinoxaline moieties plays a critical role in reinforcing the thermal resistance of the system.

Overall, Schiff base functionalization introduces localized structural heterogeneity while significantly enhancing the thermal resistance of chitosan. The observed increase in high-temperature stability and T_50_ values correlates strongly with the degree of substitution, where higher substitution levels result in enhanced thermal stability due to increased incorporation of thermally stable aromatic and imine functionalities.

#### Surface morphology investigation

**Fig. 7:**
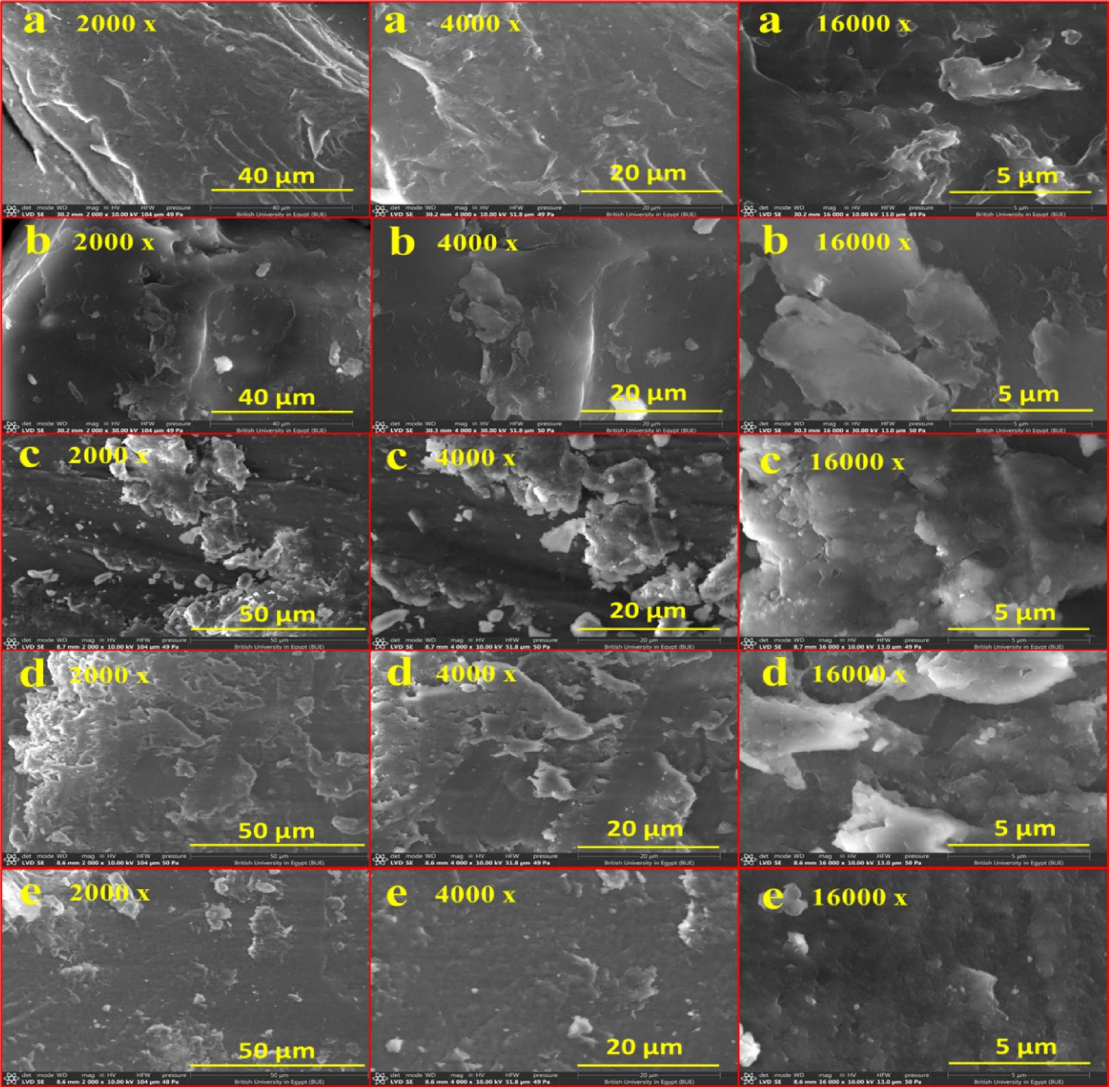
SEM micrographs of (**a**) pure chitosan and Schiff base-modified chitosan samples: (**b**) d1, (**c**) d2, (**d**) d3, and (**e**) d4 at different magnifications (2000X, 4000X, and 16000X).

SEM micrographs (Fig. 7) reveal clear morphological evolution of chitosan upon Schiff base modification. This evolution also reflects significant changes in surface topography, including variations in surface roughness, texture, and microstructural features. Native chitosan (Fig. 7a) exhibits a relatively smooth and compact surface, characteristic of its semi-crystalline nature and dense inter- and intramolecular hydrogen bonding, in good agreement with the XRD results. Even at higher magnification (16000×), only limited surface irregularities are observed, confirming its structural uniformity. In contrast, Schiff base derivatives (Fig. 7b–e) display pronounced surface alterations, including increased roughness, porosity, and heterogeneity. These changes arise from imine (C = N) bond formation, which disrupts the original hydrogen-bonded network of chitosan and introduces bulky quinoxaline substituents^56^. These modifications lead to the formation of a more irregular and topographically complex surface, which is expected to influence interfacial interactions in subsequent composite systems.

Sample d1 (highest DS, Fig. 7b) shows a comparatively smoother and more homogeneous morphology than the other derivatives, likely due to extensive surface coverage and partial pore filling by the Schiff base moieties. At intermediate substitution levels (d2 and d3; Fig. 7c,d), the surfaces become markedly rougher and more porous, reflecting non-uniform grafting and localized disruption of the polymer matrix. This morphology suggests an increased amorphous character, consistent with the reduced crystallinity observed by XRD^57^. The lowest substituted derivative (d4, Fig. 7e) retains a morphology closer to native chitosan, with fewer surface defects and limited porosity, indicating minimal structural disturbance.

Overall, the progressive increase in surface roughness and porosity with decreasing DS highlights the tunable nature of the Schiff base modification. Such topographical features are particularly advantageous for biomedical applications, as increased surface roughness and porosity are known to enhance protein adsorption, cell adhesion, and osteogenic activity, which are critical factors in bone tissue engineering.

**Fig. 8:**
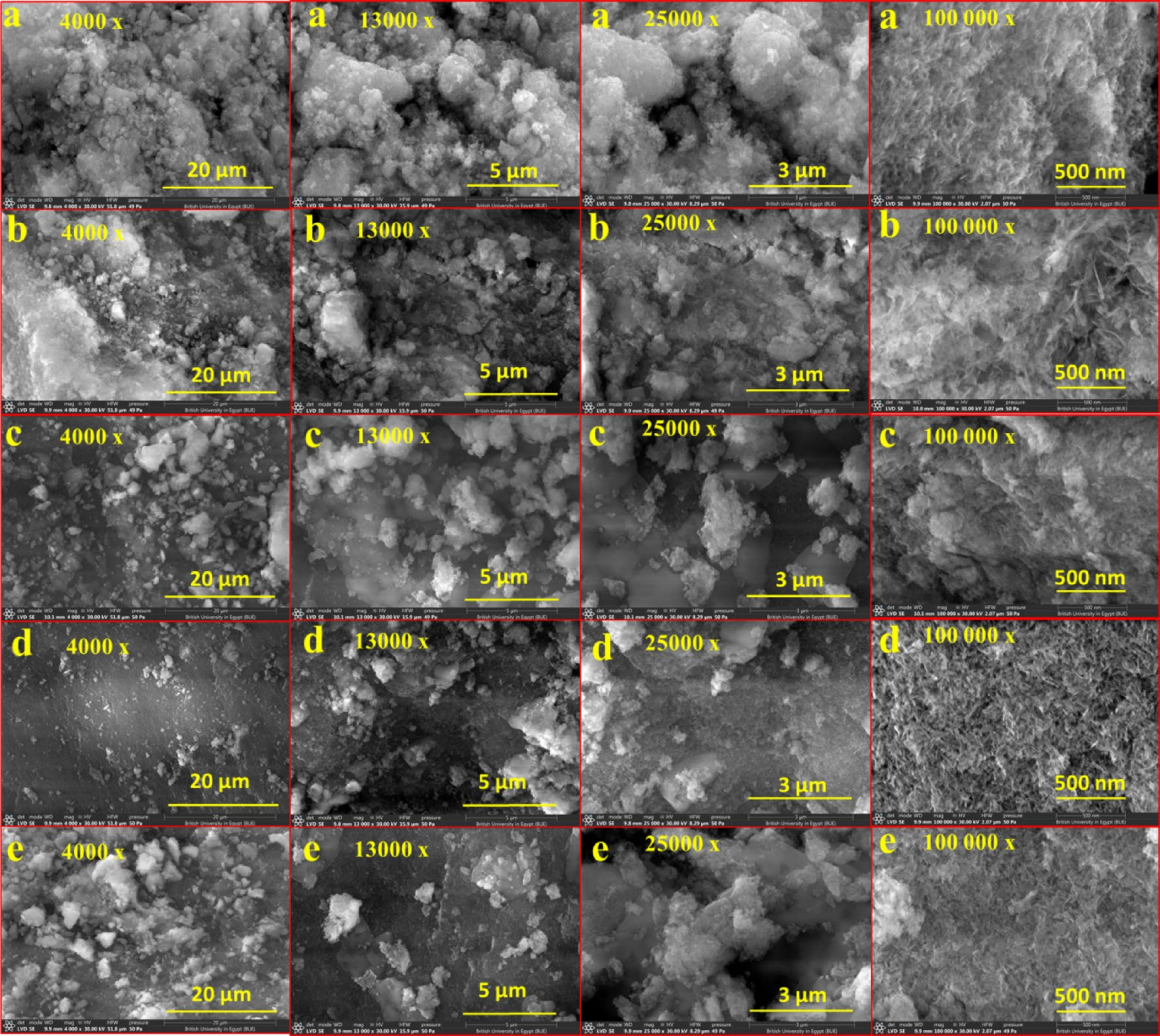
SEM micrographs of (**a**) chitosan-hydroxyapatite composite (dH0) and Schiff base-modified chitosan-hydroxyapatite composites: (**b**) dH1, (**c**) dH2, (**d**) dH3, and (**e**) dH4 at different magnifications (4000×, 13000×, 25000×, and 100000×).

The SEM micrographs in Fig. 8 reveal distinct morphological variations among the dH0–dH4 composites at magnifications of 4000× to 100,000×. Significant morphological changes were observed following Schiff base modification, influencing both the structure and potential bioactivity of the composites^58^. Unmodified composite dH0 (Fig. 8a) displayed dense agglomerates and large, irregular hydroxyapatite clusters, indicating limited dispersion and reduced surface area for biological interaction. indicating limited dispersion.

Upon Schiff base modification, the composites exhibited a progressive morphological transition toward a more porous and homogeneous microstructure. Sample dH1 showed improved dispersion and moderate roughness, suggesting enhanced interaction between the modified chitosan matrix and hydroxyapatite. The incorporation of imine and sulfonate functionalities likely facilitated electrostatic interactions with HAp, improving particle distribution. Samples dH2 and dH3 (Fig. 8c,d) exhibited the highest porosity and surface roughness, forming interconnected nanostructures with evenly distributed hydroxyapatite domains. Such interconnected and rough topography provides favorable anchoring sites for cell attachment and facilitates cell spreading and proliferation. This well-developed porous framework enhances the surface area available for bioactivity and may explain their superior antimicrobial behavior. The high-magnification images (100000×) further revealed uniform HAp nucleation sites throughout the polymer matrix, confirming strong interfacial compatibility between the Schiff base and HAp phases^59^. In contrast, dH4 (Fig. 8e), having the lowest Schiff base content, displayed smoother morphology and fewer interparticle linkages, indicating limited modification and reduced active nucleation sites. The observed morphological evolution across the series (dH0 → dH4) closely correlates with the degree of Schiff base substitution, crystallinity trends, and biological performance discussed in previous sections. Notably, dH2 exhibited the most favorable balance between porosity and structural integrity, explaining its enhanced antimicrobial efficiency and potential suitability for bone regeneration applications. This behavior highlights the critical role of surface topography in governing the biological response of the developed composites.

The N_2_ adsorption–desorption isotherm of the dH2 composite (Supplementary Fig. 7) exhibits a clear Type IV profile with an H3 hysteresis loop, confirming the presence of a mesoporous network, in accordance with IUPAC classification. The H3 loop appearing across the 0.4–1.0 P/P_0_ range is typically associated with non-rigid aggregates of plate-like particles, generating slit-shaped mesopores—a morphology consistent with the SEM observations of the composite. Textural parameters derived from BET analysis reveal a specific surface area of 44.8 m^2^ g^− 1^, accompanied by a total pore volume of 0.224 cm^3^ g^− 1^. The BJH pore size distribution (inset, Supplementary Fig. 7) displays a narrow, unimodal peak centered at ~ 20 nm, further confirming a uniform mesoporous structure. These results align with the expected porosity of chitosan–HAp hybrid systems and indicate efficient packing of the Schiff-base-modified biopolymer around HAp domains. The combination of moderate surface area, well-defined mesopores, and slit-shaped channels is particularly advantageous for biomedical applications, as it facilitates mass transport, nutrient diffusion, and adsorption of biomolecules^60^.

#### Energy Dispersive X-ray (EDX)

**Fig. 9:**
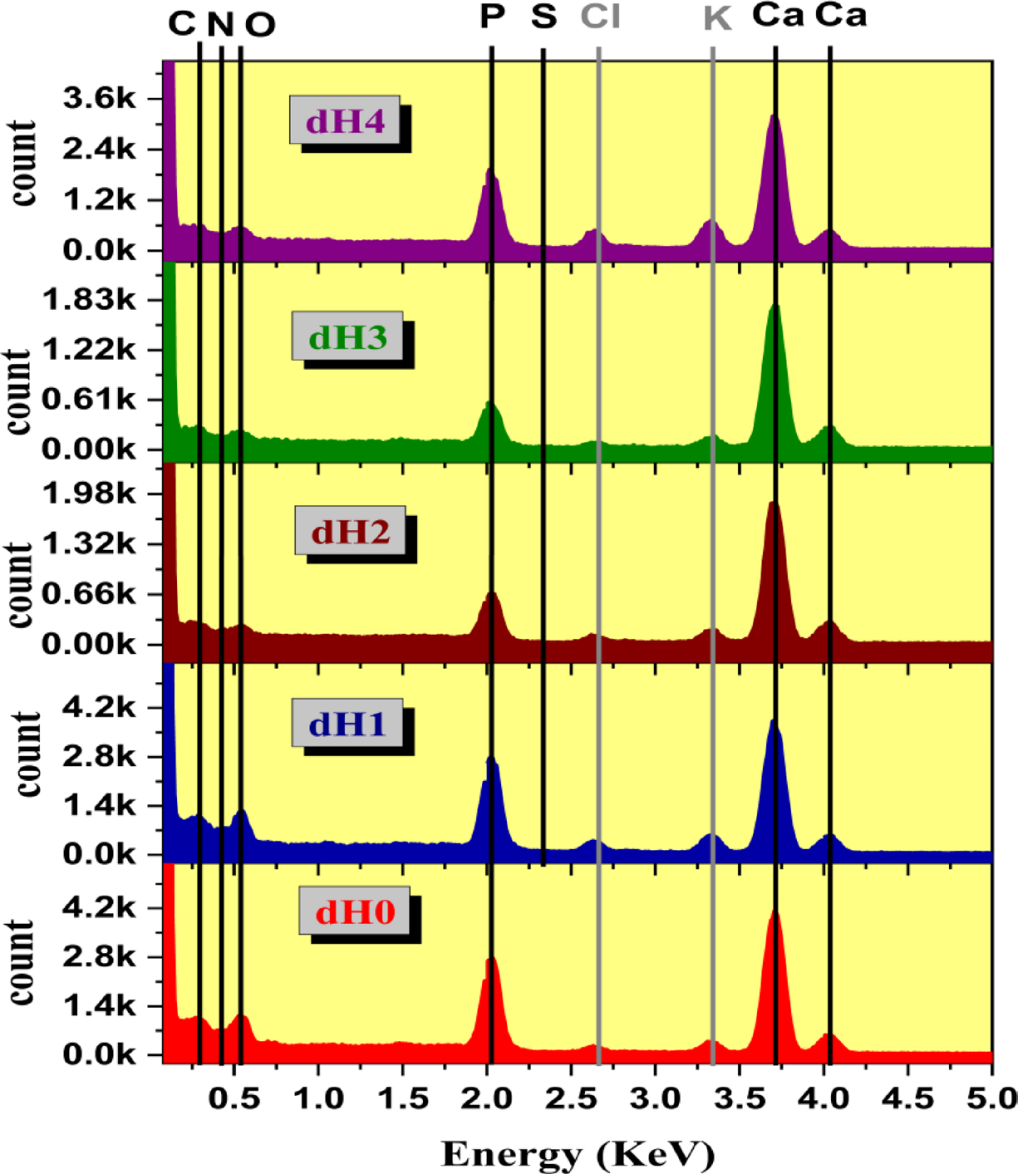
EDX spectra of hydroxyapatite composite samples (dH0, dH1, dH2, dH3 and dH4).

EDX analysis was employed to determine the elemental composition of hydroxyapatite (HAp) composites incorporating unmodified (dH0) and Schiff base–modified chitosan (dH1–dH4). The spectra (Fig. 9) and quantitative data (Supplementary Table 6) confirm the uniform incorporation of chitosan and its derivatives within the HAp matrix. Characteristic Ca and P peaks verified hydroxyapatite formation, while C and N signals originated from the chitosan backbone and Schiff base functionalities. The appearance of sulfur exclusively in dH1–dH4 provided direct evidence of the sulfonate moiety introduced through B6 functionalization. Minor Cl and K traces in all samples were attributed to residual CaCl_2_ and K_2_HPO_4_ precursors. The calculated Ca/P ratios of 1.54–1.58 were slightly below the stoichiometric value of 1.67 for pure HAp, consistent with biologically relevant, non-stoichiometric apatite’s. These deviations likely arise from partial lattice substitutions or surface complexation between HAp and Schiff base-modified chitosan, which can modulate bioactivity and dissolution behavior^61^. Collectively, EDX results confirm elemental incorporation consistent with FTIR and XRD findings. While the latter showed diminished polymer-related peaks due to HAp dominance, EDX provided complementary confirmation of the Schiff base presence, demonstrating successful chemical integration of chitosan derivatives within the HAp composite matrix.

#### Mechanical properties

The mechanical performance of the composites was assessed by comparing the reference scaffold (dH0) with the biologically optimized Schiff base–modified composite (dH2). The dH0 scaffold exhibited a compressive strength of 3.69 MPa and a Young’s modulus of 6.50 MPa, which fall within the typical range reported for chitosan/HAp scaffolds (2–12 MPa compressive strength and 2–20 MPa modulus) used in non-load-bearing bone regeneration applications^62^.

The dH2 scaffold showed a moderate reduction in compressive strength (2.97 MPa) and Young’s modulus (2.35 MPa), consistent with the softening effect commonly associated with Schiff base modification and reduced intermolecular hydrogen bonding. Importantly, these values remain within the lower but acceptable boundary reported for biopolymer-based HAp composites, ensuring that the material preserves its mechanical integrity for implantation. The strain-at-break values (19.22% for dH2 vs. 39.69% for dH0) indicate that the modification did not induce brittleness but maintained adequate elastic deformability an advantageous property for scaffolds intended for early-stage tissue ingrowth and stress dissipation^63^. Taken together, the results confirm that although Schiff base modification slightly softens the polymer matrix, the mechanical performance of dH2 remains comparable to literature-reported chitosan/HAp systems, while its significantly enhanced antimicrobial and cytocompatibility profiles offer a substantial functional advantage over unmodified chitosan scaffolds.

### Biological activity assessment

#### Antimicrobial activity of hydroxyapatite-chitosan Schiff base composites

Biofilm-associated infections remain a major limitation in hydroxyapatite-based implants. Accordingly, chitosan–HAp composites incorporating Schiff base functionalities were evaluated to determine whether chemical modification enhances antimicrobial performance compared to unmodified HAp systems^64^. The antimicrobial performance of the prepared composites (dH0–dH4) was evaluated against representative Gram-positive, Gram-negative, and fungal pathogens, with unmodified chitosan/HAp (dH0) serving as a reference.

**Fig. 10:**
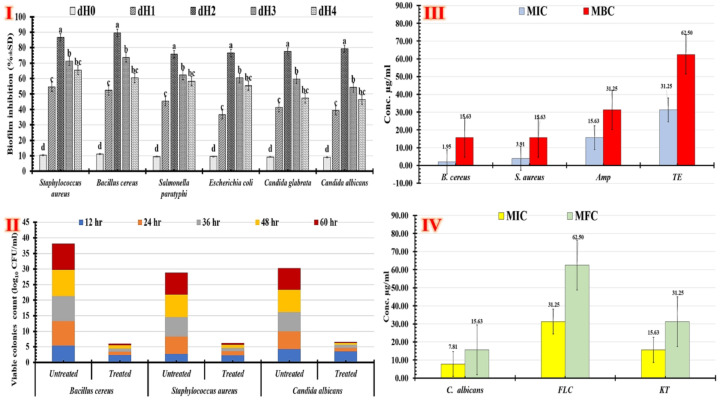
Antimicrobial performance of the HAp–chitosan Schiff base composites. (**I**) Biofilm inhibition percentages against tested human pathogens. (**II**) Time-kill kinetics of *Bacillus cereus*, *Staphylococcus aureus*, and *Candida albicans* treated with dH2 compared to untreated controls. (**III**) MIC and MBC values of dH2 against *B. cereus* and *S. aureus* compared with tetracycline (TE) and ampicillin (Amp). (**IV**) MIC and MFC values of dH2 against *C. albicans* compared with fluconazole (FLC) and ketoconazole (KT).

Biofilm inhibition results (Table 4; Fig. 10I) demonstrate a clear enhancement in antimicrobial efficacy upon Schiff base modification. Among all tested formulations, dH2 exhibited the highest biofilm inhibition against both Gram-positive and Gram-negative bacteria as well as yeast strains. Tukey’s post hoc test confirmed these differences at a 95% confidence level (*p* ≤ 0.05). The mean difference between dH2 and control groups was statistically significant. The dH2 formulation achieved the highest biofilm inhibition against *Bacillus cereus* (89.65 ± 1.22%) and *Staphylococcus aureus* (86.65 ± 3.36%). In contrast, lower yet comparable inhibition rates were observed for *Candida albicans* (79.29 ± 1.54%) and *Candida glabrata* (77.52 ± 1.98%). The lowest inhibition percentages were recorded against gram-negative bacteria, with *Escherichia coli* at 76.54 ± 1.08% and *Salmonella paratyphi* at 75.84 ± 0.98%.

**Table 4:** Shows that all formulations exhibited varying degrees of biofilm inhibition across the tested pathogens.

Human pathogens	Biofilm inhibition (%±SD)				
	dH0	dH1	dH2	dH3	dH4
*Staphylococcus aureus*	10.38 ± 0.83^d^	54.63 ± 5.23^c^	86.65 ± 3.36^a^	71.32 ± 2.98^b^	65.54 ± 2.09^bc^
*Bacillus cereus*	11.16 ± 0.32^d^	52.33 ± 1.14^c^	89.65 ± 1.22^a^	73.66 ± 1.11^b^	60.32 ± 2.35^bc^
*Salmonella paratyphi*	9.48 ± 0.54^d^	45.32 ± 0.98^c^	75.84 ± 0.98^a^	62.33 ± 2.35^b^	58.32 ± 0.98^bc^
*Escherichia coli*	9.56 ± 1.22^d^	36.65 ± 1.87^c^	76.54 ± 1.08^a^	60.32 ± 2.87^b^	55.41 ± 1.58^bc^
*Candida glabrata*	9.28 ± 1.34^d^	41.32 ± 1.06^c^	77.52 ± 1.97^a^	59.65 ± 0.58^b^	47.36 ± 2.54^bc^
*Candida albicans*	9.04 ± 0.69^d^	39.33 ± 0.87^c^	79.29 ± 1.54^a^	54.32 ± 0.98^b^	46.35 ± 1.87^bc^

The data is shown as the mean percentage± standard deviation (%±SD). The differences in the superscript letters are statistically significant at *p* < 0.05. R-sq (94.45%), adj R-sq (93.56%), and pred R-sq (92.00%).

The time-kill kinetics for all chosen human pathogens were examined in this work in order to better investigate the antimicrobial abilities of dH2-formula. Supplementary Table 7 summarizes the reported time-kill kinetics findings. Figure 10II shows a stacked bar graph illustrating the reduction of planktonic pathogen viability by the dH2 formula compared to untreated controls. After 60 h incubation period with dH2-formula, the growth reduction percentages of *Bacillus cereus*, *Candida albicans*, and *Staphylococcus aureus* increased to 93.81 ± 0.68, 92.53 ± 0.11, and 91.03 ± 0.84%, respectively. The studied antimicrobial agent’s critical duration, which would reduce the initial inoculum by > 3 log_10_ CFU/ml, was identified to be the bactericidal time (99.9% killing)^65^. After 72 h of incubation, the dH2-formula achieved complete biofilm suppression of all tested pathogens, exceeding 0.52 log_10_ CFU/mL reduction. Complete eradication (≥ 99.9% killing) of biofilm-forming microorganisms was achieved within 120 h, indicating sustained bactericidal and fungicidal activity^66,67^.

The minimum inhibitory concentrations (MICs) of the dH2 composite against *Bacillus cereus* and *Staphylococcus aureus* were determined to be 1.95 and 3.91 µg/mL, respectively (Fig. 10III). These values are markedly lower than those of the reference antibiotics ampicillin (15.625 µg/mL) and tetracycline (31.25 µg/mL), indicating the superior antimicrobial efficiency of the developed composite. For *Candida albicans*, the MIC value was 7.81 µg/mL (Fig. 10IV), which was significantly lower than those recorded for ketoconazole (15.63 µg/mL) and fluconazole (31.25 µg/mL). Moreover, the minimum bactericidal/fungicidal concentrations (MBC/MFC) required to achieve ≥ 99.99% microbial killing for all tested strains were uniformly measured at 15.63 µg/mL (Fig. 10III and IV). In contrast, the corresponding bactericidal concentrations of the tested antibiotics ranged from 31.25 to 62.50 µg/mL. These results clearly demonstrate that the dH2 composite exhibits potent antimicrobial activity at significantly lower concentrations than conventional antimicrobial agents. The enhanced antimicrobial performance of dH2 can be attributed to the synergistic effects of the chitosan matrix, Schiff base functionality, and hydroxyapatite.

Previous studies have demonstrated the antimicrobial potential of chitosan- and hydroxyapatite-based systems against a broad spectrum of clinical pathogens. For instance, chitosan/HAp nanocomposites and vitamin K_2_-containing formulations showed pronounced activity against *Staphylococcus aureus* and *Candida albicans*, which were among the most sensitive strains reported^68^. Similarly, PVP/chitosan/HAp scaffolds incorporated with silver nanoparticles exhibited effective antibacterial performance against *E. coli*, *P. aeruginosa*, *K. pneumoniae*, and *S. aureus*^69^. Antibiotic-loaded HAp–chitosan systems, such as ciprofloxacin-containing 3D composites, also demonstrated relevant antibacterial activity against *E. coli* and *S. aureus* in orthopedic applications^70^. Moreover, porous HAp/chitosan scaffolds were found to be particularly effective against Gram-positive bacteria, achieving bacterial reductions exceeding 90% for *S. aureus*, while showing comparatively lower activity against Gram-negative strains^71^. In comparison, the present dH2 composite achieves comparable or superior antimicrobial efficacy without the incorporation of external antibiotics or metal nanoparticles, highlighting the advantage of Schiff base functionalization in imparting intrinsic antimicrobial activity.

#### In vitro cytotoxicity evaluation

The cytocompatibility of the dH2 composite was evaluated using two cell models to obtain a comprehensive assessment of its biological safety: Vero cells for preliminary toxicity screening and MG-63 osteoblast-like cells to determine bone-related biocompatibility. Viability data for both models at concentrations ranging from 31.25 to 1000 µg/mL are presented in Supplementary Table 8 (Vero) and 9 (MG-63). In Vero cells, viability decreased sharply to ~ 2.2% at 1000 µg/mL but remained above 99% at concentrations ≤ 125 µg/mL. The calculated IC₅₀ value (344.99 ± 2.55 µg/mL) indicates a favorable safety profile at lower concentrations. MG-63 cells exhibited a similar dose-dependent trend but with higher sensitivity, consistent with their osteoblastic nature. Viability remained ~ 100% at 31.25 µg/mL and 97% at 62.5 µg/mL, while decreasing significantly at higher concentrations. This observation is consistent with the cancer-derived origin of MG-63 cells, which are generally more sensitive to external stimuli compared to normal cell lines, and therefore provide a complementary model for evaluating cytocompatibility. The half-maximal inhibitory concentration (IC_50_) value for MG-63 cells (156.62 ± 1.63 µg/mL) indicates acceptable cytocompatibility within the tested concentration range relevant for antibacterial performance^72^. Importantly, the viability region above 90% overlaps with concentrations that produced strong antimicrobial activity, suggesting a favorable therapeutic window under the tested conditions.

**Fig. 11:**
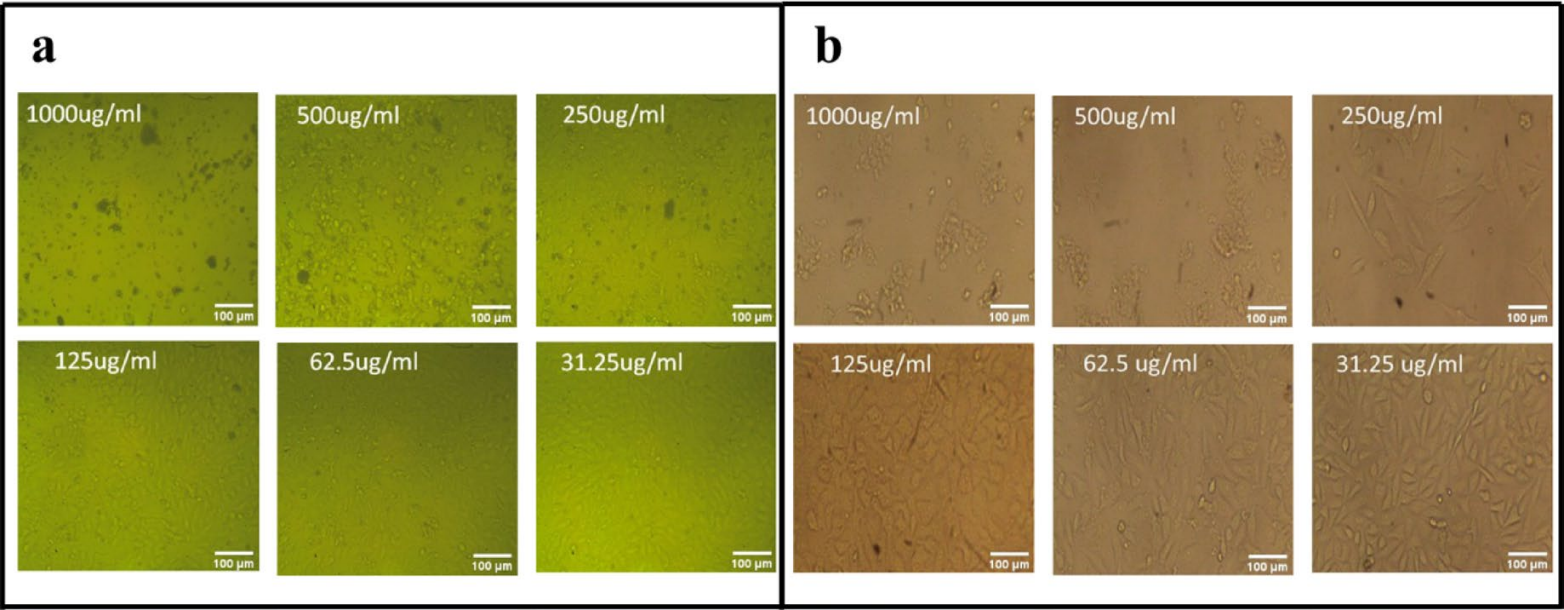
Representative micrographs of (**a**) Vero cells and (**b**) MG-63 osteoblast-like cells treated with dH2 at different concentrations (1000–31.25 µg/mL), as indicated within each panel.

Morphological assessment corroborated the quantitative data: normal cell morphology was preserved at low concentrations, whereas higher doses induced typical signs of cytotoxicity, including rounding and partial monolayer loss (Fig. 11a,b). At lower concentrations, MG-63 cells exhibited a well-spread and elongated morphology with clear cell–cell contact, indicating favorable cell adherence and strong interaction with the composite surface. The absence of cell detachment or shrinkage under these conditions further supports the cytocompatibility and suitability of the material for cell anchorage. Overall, the combined results from Vero and MG-63 cells suggest that the dH2 composite maintains a favorable safety–efficacy balance under the tested conditions. Notably, antibacterial activity was achieved at concentrations that remained non-toxic to both normal mammalian and osteoblast-like cells. Furthermore, the ability of MG-63 cells to maintain normal morphology and adherence suggests a favorable microenvironment for osteogenic activity. Combined with the presence of well-defined hydroxyapatite phases confirmed by XRD and Ca/P ratios obtained from EDX analysis, these findings indirectly support the biomineralization potential of the developed composite.

## Conclusions

A new quinoxaline-based chitosan Schiff base/hydroxyapatite composite was successfully synthesized and evaluated as an antimicrobial biomaterial for bone regeneration. Covalent grafting of the sulfonated quinoxaline aldehyde (B6) onto chitosan enabled precise control over the degree of substitution. This parameter governed crystallinity, ion-exchange capacity, solubility behavior, thermal stability, and surface morphology. Structural analyses (FTIR, NMR, XRD and XPS) confirmed imine bond formation and strong organic–inorganic interfacial interactions. Scanning electron microscopy with energy-dispersive X-ray analysis (SEM–EDX) and BET analysis demonstrated homogeneous hydroxyapatite integration and a well-developed mesoporous architecture. Among the investigated formulations, the dH2 composite emerged as the optimal candidate. It exhibited a favorable balance between structural integrity, controlled porosity, and interfacial compatibility. This optimized structure was associated with improved biological performance. The dH2 composite demonstrated notable antimicrobial activity against Gram-positive bacteria, Gram-negative bacteria, and yeast, achieving biofilm inhibition values approaching 90% and exhibiting MIC/MBC values lower than those of the reference antimicrobial agents under the tested in vitro conditions. Importantly, dH2 maintained good cytocompatibility toward both Vero and MG-63 osteoblast-like cells within the effective antibacterial concentration range. Mechanical evaluation further indicated that the dH2 composite retained acceptable compressive strength and elastic deformability within the range reported for non-load-bearing chitosan/HAp-based scaffolds. Overall, these findings demonstrate that quinoxaline-based Schiff base functionalization is a promising strategy for imparting intrinsic antimicrobial activity to chitosan–hydroxyapatite systems. Moreover, this modification does not compromise the composites’ biocompatibility. Additionally, the structural performance of the materials remains intact, highlighting their potential for biomedical applications. The developed dH2 composite represents a promising platform for the development of antimicrobial bone scaffolds and implant coatings. However, the present study is limited to in vitro evaluation. Further investigations—including cell adhesion, biomineralization, and in vivo studies—are required to confirm the long-term performance and clinical applicability of the developed composite.

## Supplementary Information

Below is the link to the electronic supplementary material.

Supplementary Material 1

Supplementary Material 2

Supplementary Material 3

## Data Availability

All data generated or analyzed during this study are included in this published article and its supplementary information files.
