# Supplementary material for: Chitosan-quinoxaline Schiff base hydroxyapatite composite with antimicrobial properties for bone regeneration
Source: Sci Rep. 2026 Jul 15;16:22252. doi: 10.1038/s41598-026-61289-w (PMC13373163; doi:10.1038/s41598-026-61289-w)
Supplement: Supplementary file 2 — Supplementary Material 2 [file 41598_2026_61289_MOESM2_ESM.docx]

**Chitosan-Quinoxaline Schiff Base Hydroxyapatite Composite with Antimicrobial Properties for Bone Regeneration**

**Supplementary data**

**Sup. Table 1**. Nitrogen content and degree of substitution (DS) for Schiff base derivatives (d1–d4) synthesized with varying B6 ratios.

| Sample Code | Nitrogen Content (%) | Degree of Substitution (DS) |
| --- | --- | --- |
| d1 | 7.45% | 0.631 |
| d2 | 7.30% | 0.623 |
| d3 | 7.10% | 0.613 |
| d4 | 6.90% | 0.602 |

**Sup. Table 2.** Crystallinity index (CI) values of chitosan and its Schiff base derivatives.

| Sample | CI (%) |
| --- | --- |
| Chitosan | **67** |
| d1 | **48.5** |
| d2 | **53** |
| d3 | **56.7** |
| d4 | **59.5** |

**Sup. Table 3.** XRD-derived crystallite size data for HAp composites (dH0–dH4).

| Sample | 2θ (°) | FWHM (°2θ) | Crystallite size D (nm) | Average crystallite size (nm) |
| --- | --- | --- | --- | --- |
| dH0 | 25.88 | 0.44 | 18.49 | **9.97** |
|  | 32.06 | 1.47 | 5.64 |  |
|  | 28.47 | 1.08 | 7.56 |  |
|  | 33.99 | 0.89 | 9.28 |  |
|  | 39.67 | 1.16 | 7.30 |  |
|  | 46.72 | 0.75 | 11.56 |  |
|  | 49.67 | 1.55 | 5.66 |  |
|  | 53.19 | 0.32 | 27.79 |  |
| dH1 | 25.87 | 0.44 | 18.69 | **18.21** |
|  | 28.32 | 0.38 | 21.80 |  |
|  | 32.11 | 1.70 | 4.85 |  |
|  | 40.50 | 0.36 | 23.64 |  |
|  | 46.66 | 1.13 | 7.68 |  |
|  | 53.23 | 0.27 | 32.56 |  |
|  | 49.67 | 2.05 | 4.27 |  |
| dH2 | 25.88 | 0.69 | 11.82 | **12.90** |
|  | 28.32 | 0.37 | 21.94 |  |
|  | 31.91 | 1.46 | 5.65 |  |
|  | 33.40 | 1.53 | 5.41 |  |
|  | 40.51 | 0.32 | 26.76 |  |
|  | 46.98 | 1.48 | 5.84 |  |
|  | 49.94 | 2.01 | 4.36 |  |
|  | 53.28 | 0.30 | 29.23 |  |
| dH3 | 25.87 | 0.46 | 17.62 | **14.37** |
|  | 28.36 | 0.48 | 17.19 |  |
|  | 32.00 | 1.34 | 6.15 |  |
|  | 33.45 | 1.75 | 4.73 |  |
|  | 40.54 | 0.28 | 30.79 |  |
|  | 46.68 | 0.89 | 9.74 |  |
|  | 49.66 | 2.27 | 3.85 |  |
| dH4 | 25.83 | 0.74 | 11.05 | **10.89** |
|  | 28.34 | 0.77 | 10.70 |  |
|  | 31.95 | 1.69 | 4.90 |  |
|  | 33.82 | 1.31 | 6.32 |  |
|  | 40.50 | 0.28 | 30.12 |  |
|  | 48.98 | 3.85 | 2.27 |  |

**Sup. Table 4.** Crystallographic parameters of HAp–chitosan Schiff base composites.

| Sample | Crystal system | a (Å) | b (Å) | c (Å) | α (°) | β (°) | γ (°) | Unit cell volume (Å³) | Z | RIR |
| --- | --- | --- | --- | --- | --- | --- | --- | --- | --- | --- |
| **dH0** | **Hexagonal** | **3.8911** | **3.8911** | **13.1863** | **90** | **90** | **120** | **172.90** | **2** | **3.25** |
| **dH1** | **Hexagonal** | **3.8911** | **3.8911** | **13.1863** | **90** | **90** | **120** | **172.90** | **2** | **3.25** |
| **dH2** | **Hexagonal** | **3.6290** | **3.6290** | **5.8900** | **90** | **90** | **120** | **67.18** | **1** | **1.93** |
| **dH3** | **Hexagonal** | **3.8911** | **3.8911** | **13.1863** | **90** | **90** | **120** | **172.90** | **2** | **3.25** |
| **dH4** | **Hexagonal** | **3.8911** | **3.8911** | **13.1863** | **90** | **90** | **120** | **172.90** | **2** | **3.25** |

**Sup. Table 5.** Binding energies and chemical assignments of XPS core-level peaks for samples d2, dH0, and dH2.

| Sample | Element | Core level | Binding energy (eV) | Peak assignment |
| --- | --- | --- | --- | --- |
| d2 | C | C1s | 285.37 | C–C / C–H |
|  | C | C1s | 286.74 | C–O / C–N |
|  | C | C1s | 287.95 | C=N / C=O (Schiff base) |
|  | N | N1s | 397.28 | Imine nitrogen (C=N) |
|  | N | N1s | 400.19 | Free amine nitrogen |
|  | N | N1s | 402.99 | Protonated nitrogen |
|  | O | O1s | 531.63 | Carbonyl / sulfonyl oxygen |
|  | O | O1s | 532.60 | Ether / hydroxyl oxygen |
|  | S | S2p | 163.41–164.50 | Sulfonated quinoxaline sulfur |
| dH0 | C | C1s | 285.52 | C–C / C–H |
|  | C | C1s | 286.00–286.61 | C–O / C–N |
|  | N | N1s | 400.01–400.55 | Amine nitrogen |
|  | O | O1s | 531.86 | Phosphate oxygen (PO₄³⁻) |
|  | Ca | Ca2p | 345.88 / 349.62 | Ca²⁺ in hydroxyapatite |
|  | P | P2p | 130.78 / 131.55 | Phosphate (PO₄³⁻) |
| dH2 | C | C1s | 285.64 | C–C / C–H |
|  | C | C1s | 286.55 | C–O / C–N |
|  | N | N1s | 400.00 | Amine nitrogen |
|  | N | N1s | 401.49 | Imine nitrogen (retained Schiff base) |
|  | O | O1s | 531.00 | Phosphate oxygen |
|  | O | O1s | 529.32 | Ca–O coordinated oxygen |
|  | Ca | Ca2p | 345.90 / 349.57 | Ca²⁺ in hydroxyapatite |
|  | P | P2p | 130.76 / 131.51 | Phosphate (PO₄³⁻) |
|  | S | S2p | 163.22 / 166.99 | Sulfonated quinoxaline sulfur |

**Sup. Table 6.** Elemental composition (At% and Wt%) and Ca/P ratio of hydroxyapatite composites (dH0–dH4) as determined by EDX analysis.

| Sample | C | | N | | O | | P | | Ca | | S | | Cl | | K | | Ca/P  ratio |
| --- | --- | --- | --- | --- | --- | --- | --- | --- | --- | --- | --- | --- | --- | --- | --- | --- | --- |
|  | At% | Wt% | At% | Wt% | At% | Wt% | At% | Wt% | At% | Wt% | At% | Wt% | At% | Wt% | At% | Wt% |  |
| dH0 | **10.73** | **8.93** | **6.8** | **6.12** | **8.41** | **7.32** | **27.75** | **23.88** | **43.6** | **48.21** | **_** | **_** | **2.26** | **2.75** | **3.45** | **2.79** | **1.57** |
| dH1 | **11.2** | **9.45** | **6.5** | **5.86** | **8.25** | **7.19** | **27.12** | **23.33** | **41.7** | **46.21** | **1.45** | **1.31** | **3.02** | **2.89** | **4.76** | **3.89** | **1.54** |
| dH2 | **6.34** | **5.45** | **4.25** | **3.88** | **6.58** | **5.72** | **30.25** | **26.11** | **47.75** | **51.28** | **0.94** | **0.86** | **3.12** | **2.91** | **4.77** | **3.92** | **1.58** |
| dH3 | **8.21** | **7.12** | **5.12** | **4.71** | **6.98** | **6.07** | **29.5** | **25.44** | **46.3** | **49.89** | **1.02** | **0.94** | **2.65** | **2.53** | **3.92** | **3.24** | **1.57** |
| dH4 | **9.65** | **8.43** | **6.21** | **5.72** | **7.45** | **6.48** | **28.75** | **24.78** | **44.8** | **48.36** | **1.23** | **1.12** | **3.87** | **3.64** | **5.04** | **4.15** | **1.56** |

**Sup. Table 7.** In vitro study for determining time-kill kinetics of dH2-treated *Bacillus cereus, Staphylococcus aureus*, and *Candida albicans* with their untreated controls.

| Incubation period (h) | Human pathogens treated with dH2-formula | | | | | | | | |
| --- | --- | --- | --- | --- | --- | --- | --- | --- | --- |
|  | ***Bacillus cereus*** | | | ***Staphylococcus aureus*** | | | ***Candida albicans*** | | |
|  | **Cell viability (log_10_CFU/ml±SD)** | | **Percentage of reduction in growth** | **Cell viability (log_10_CFU/ml±SD)** | | **Percentage of reduction in growth** | **Cell viability (log_10_CFU/ml±SD)** | | **Percentage of reduction in growth** |
|  | **Untreated** | **Treated** | **(%±SD)** | Untreated | Treated | **(%±SD)** | Untreated | Treated | **(%±SD)** |
| 12 | 5.36±0.35 | 2.36±0.36 | 55.97±0.14 | 2.71±0.35 | 2.31±1.25 | 14.76±0.14 | 4.31±2.11 | 3.52±0.98 | 18.32±0.94 |
| 24 | 7.94±1.23 | 1.12±1.25 | 85.89±0.42 | 5.63±1.22 | 1.32±0.88 | 76.55±0.41 | 5.63±0.24 | 1.14±0.02 | 79.75±0.13 |
| 36 | 8.02±0.65 | 1.04±0.98 | 87.03±2.41 | 6.24±0.87 | 1.02±0.04 | 83.65±0.38 | 6.24±0.47 | 0.97±0.03 | 84.45±0.51 |
| 48 | 8.45±1.52 | 0.96±0.21 | 88.63±0.90 | 7.22±0.33 | 0.97±0.21 | 86.56±0.50 | 7.11±1.24 | 0.54±0.11 | 92.40±0.63 |
| 60 | 8.41±2.54 | 0.52±0.14 | 93.81±0.68 | 7.03±2.05 | 0.63±0.05 | 91.03±0.84 | 6.98±0.98 | 0.42±0.09 | 92.53±0.11 |

**Sup. Table 8.** Cell viability percentages of Vero cells after exposure to different concentrations of the dH2 composite, evaluated using the MTT assay.

| ID | ug/ml | O.D | | | Mean O.D | ±SE | Viability % | Toxicity % | IC50  ± SD |
| --- | --- | --- | --- | --- | --- | --- | --- | --- | --- |
| Vero | -------- | 0.761 | 0.794 | 0.788 | 0.781 | 0.010149 | 100 | 0 | ug |
| dH2 | 1000 | 0.018 | 0.019 | 0.015 | 0.017333 | 0.001202 | 2.219376867 | 97.78062313 | 344.99 ± 2.55 |
|  | 500 | 0.115 | 0.139 | 0.126 | 0.126667 | 0.006936 | 16.21852326 | 83.78147674 |  |
|  | 250 | 0.537 | 0.531 | 0.568 | 0.545333 | 0.011465 | 69.82501067 | 30.17498933 |  |
|  | 125 | 0.769 | 0.773 | 0.785 | 0.775667 | 0.004807 | 99.31711481 | 0.68288519 |  |
|  | 62.5 | 0.783 | 0.762 | 0.769 | 0.771333 | 0.006173 | 98.76227059 | 1.237729407 |  |
|  | 31.25 | 0.77 | 0.79 | 0.782 | 0.780667 | 0.005812 | 99.95731968 | 0.042680324 |  |

**Sup. Table 9.** Cell viability (%), optical density, and IC₅₀ values of MG-63 osteoblast-like cells treated with dH2 at different concentrations (31.25–1000 µg/mL).

| **ID** | **ug/ml** | **O.D** | | | **Mean O.D** | **±SE** | **Viability %** | **Toxicity %** | **IC50**  **± SD** |
| --- | --- | --- | --- | --- | --- | --- | --- | --- | --- |
| MG63 | -------- | 0.688 | 0.691 | 0.685 | 0.688 | 0.001732 | 100 | 0 | ug |
| dH2 | 1000 | 0.02 | 0.019 | 0.022 | 0.020333 | 0.000882 | 2.955426357 | 97.04457364 | 156.62 ± 1.63 |
|  | 500 | 0.02 | 0.02 | 0.021 | 0.020333 | 0.000333 | 2.955426357 | 97.04457364 |  |
|  | 250 | 0.094 | 0.108 | 0.1 | 0.100667 | 0.004055 | 14.63178295 | 85.36821705 |  |
|  | 125 | 0.354 | 0.369 | 0.363 | 0.362 | 0.004359 | 52.61627907 | 47.38372093 |  |
|  | 62.5 | 0.662 | 0.668 | 0.675 | 0.668333 | 0.003756 | 97.14147287 | 2.858527132 |  |
|  | 31.25 | 0.685 | 0.689 | 0.69 | 0.688 | 0.001528 | 100 | 0 |  |


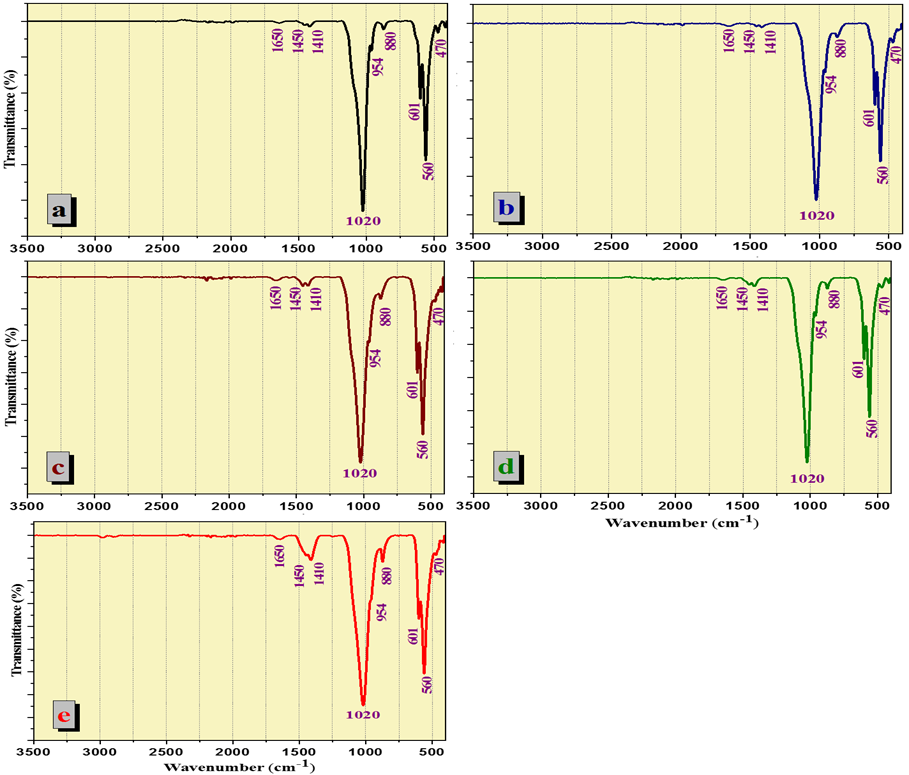


**Sup. Figure 1.** FTIR spectra of (a) dH0 sample, (b) dH1 sample, (c) dH2 sample, (d) dH3 sample and (e) dH4 sample.

**a**

**b**

**c**

**Sup. Figure 2.** High-resolution C1s XPS spectra of (a) d2, (b) dH0, and (c) dH2.

**a**

**b**

**c**

**Sup. Figure 3.** High-resolution N1s XPS spectra of (a) d2, (b) dH0, and (c) dH2.

**a**

**b**

**c**

**Sup. Figure 4.** High-resolution O1s XPS spectra of (a) d2, (b) dH0, and (c) dH2.

**a**

**b**

**Sup. Figure 5.** High-resolution Ca2p XPS spectra of (a) dH0 and (b) dH2.

**a**

**b**

**Sup. Figure 6.** High-resolution P2p XPS spectra of (a) dH0 and (b) dH2.


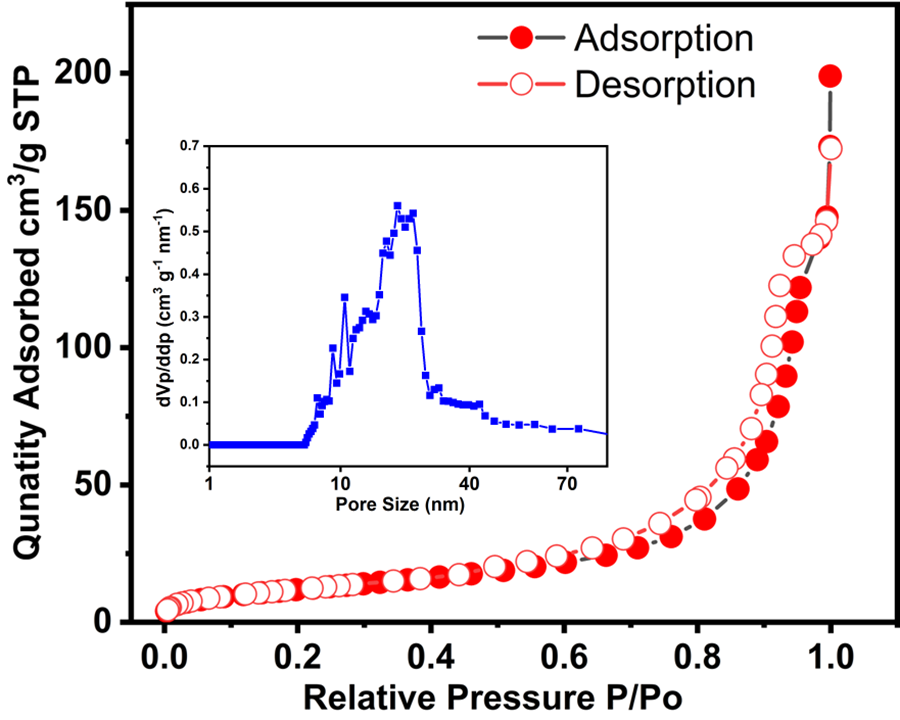


**Sup. Figure 7.** N_2_ adsorption-desorption allowing surface area calculation via BET method and Pore size distribution curve for dh2 sample.
